# A microfluidic hollow-fiber infection model (µHFIM): monitoring bacterial response to dynamic drug treatment with single-cell resolution

**DOI:** 10.1038/s41378-026-01377-z

**Published:** 2026-07-09

**Authors:** Friederike-Leonie Born, Raphael Dezauzier, Annelies S. Zinkernagel, Petra S. Dittrich

**Affiliations:** 1 Department of Biosystems Science and Engineering, ETH Zurich, Basel, Switzerland; 2 Department of Infectious Diseases and Hospital Epidemiology, University Hospital Zurich, University of Zurich, Zurich, Switzerland

**Keywords:** Engineering, Microfluidics

## Abstract

Antibiotic resistance is a growing global health threat. To improve our understanding of the mechanisms driving antibiotic resistance, suitable infection models are needed that also capture relevant drug dynamics. Here, we present a microfluidic hollow-fiber infection model that enables pharmacokinetic/pharmacodynamic studies under physiologically relevant conditions. The system simulates temporally antibiotic concentration gradients (pharmacokinetic module) and enables the long-term, high-resolution imaging of bacterial cells within a tissue-like hydrogel matrix in a flat growth chamber (pharmacodynamic module) with minimum sample and drug consumption. Using *Escherichia coli* quality control strains exposed to amoxicillin and amoxicillin-clavulanic acid, we show that treatment efficacy is not determined solely by the fraction of time above the minimum inhibitory concentration. Instead, dosing intervals and recovery phases between doses critically shape bacterial survival and killing. Prolonged dosing phases (>6 h) or shortened recovery periods enhance bacterial clearance, while specific dynamic regimens induce distinct phenotypic responses, including filamentous growth. In addition, the system supports analysis of clinical isolates via inline staining. By combining dynamic PK control, tissue-like hydrogel environments, and sustained high-resolution imaging, the µHFIM enables in vivo-like investigation of bacterial responses to antibiotic treatment. This system provides mechanistic insight into how dosing schedules govern efficacy and adaptation, which may help to optimize antibiotic treatment strategies in the future (bench to bedside translation).

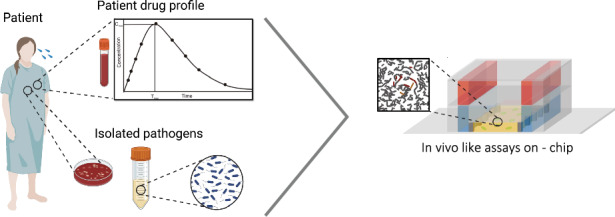

## Introduction

Antimicrobial resistance (AMR) is a serious global crisis threatening human and animal health^1,2^. In 2021, 4.71 million deaths were associated with bacterial AMR in the world, including 1.14 million directly attributable to bacterial AMR^3^. While resistance is rising, the development of new antibiotics is declining, and no effective combating strategy is available^4^. In addition to developing new drugs, optimizing the use of existing antibiotics is crucial^5^. Potent in vitro models are needed to understand the different mechanisms of resistance and persistence in bacteria when exposed to drugs and drug candidates.

Resistant bacteria replicate in the presence of a drug, whereas persistent bacteria survive exposure to a bactericidal drug concentration without replicating^6^. Drug potency is commonly measured by the minimum inhibitory concentration (MIC), which is the lowest concentration of the drug that prevents bacterial replication^6^. The MIC is a key parameter in preclinical studies^7^. Routine methods for characterizing antibiotic resistance in clinical isolates are in vitro static assays, such as disc diffusion, broth microdilution, standardized by EUCAST and CLSI^8,9^, and automated systems like Vitek^10,11^. However, these methods do not reflect dynamic drug concentrations in vivo, fail to monitor morphological changes over time, and cannot detect persister cells through regrowth analysis after drug removal.

In the human body, antibiotic concentrations fluctuate over time due to absorption, distribution, metabolism, and excretion, creating temporal antibiotic gradients from the transient peaks and troughs of drug exposure. Recent studies show that spatial^12,13^ and temporal^14–16^ variations in drug levels can significantly influence resistance development. Therefore, models should be capable of recapitulating the pharmacokinetics (PK) profile of a drug in the human body and of monitoring the drug’s impact on the growth and survival of the bacteria (referred to as pharmacodynamics, PD). While PK parameters quantify the time course of an antibiotic, they do not describe the lethal effect of an antibiotic drug. The three most important PK parameters for evaluating antibiotic efficacy are peak concentration (*c*_max_), trough concentration (*c*_min_), and area under the serum concentration time curve (AUC). PK parameters integrated with the MIC give the PK/PD indices, which quantify the activity of an antibiotic. These PK/PD indices are the percent of time the free drug concentration remains above the MIC (%fT > MIC), the ratio of the free drug peak concentration to the MIC (f *c*_max_:MIC), and the ratio of the area under the free drug concentration time-curve to the MIC (fAUC:MIC)^17^.

Current in vitro PK/PD models use one or more compartments to culture the cells while delivering drugs at defined concentrations^18,19^. The most widely used system is the hollow-fiber infection model (HFIM), which supports drug sampling, kinetic monitoring, and testing of drug combinations^20–22^. The HFIMs require large volumes of media, drugs, and cells, leading to high running costs, and furthermore, the invasive sampling represents only a finite time point measured as an average of the entire population. The HFIM cannot be coupled with time-lapse microscopy and thus cannot investigate individual cell behavior over time. Heterogeneity within bacterial populations is known to play a key role in antibiotic resistance and persistence and typically evolves due to a manifold of biological and environmental factors. Rare phenotypes may influence the entire population’s fate with respect to growth or infection, especially under fluctuating environmental conditions^23^. These cells are challenging to detect during conventional analysis. Hence, spatial and temporal resolution of the model is essential^24^.

In the past decade, microfluidic devices have enabled the cultivation of bacterial cells inside well-controllable environments, allowing for continuous monitoring of their behavior over time using time-lapse microscopy. Since many bacteria are highly motile, they need to be confined in microfluidic chambers. This has been achieved by (i) enclosing the chamber with membranes^25–29^, (ii) physically trapping bacteria in flat chambers^30^ or narrow and flat channels (e.g., “mother machine”^31,32^), which supports 2D growth and facilitates single-cell imaging, and (iii) embedding cells in hydrogels confined by pillar arrays, e.g., used for chemotactic studies^28,33–35^. In the context of drug testing, such microfluidic devices were frequently used for antibiotic susceptibility testing, where a constant drug concentration is administered and cell response is assessed by monitoring cell growth. Kim et al. also used this hydrogel approach to monitor *P. aeruginosa* under spatial antibiotic gradients and estimated bacterial densities^36^.

In this study, we combine the benefits of miniaturization for capturing and monitoring bacterial cells in defined compartments with the goal to develop a system capable of understanding the effect of *dynamic* drug exposure akin to the HFIM. Therefore, we refer to our system as the microfluidic hollow-fiber infection model (µHFIM). In contrast to the previous microfluidic systems, the here introduced µHFIM facilitates the precise administration of temporal and clinically relevant antibiotic concentration cycles and long-term studies over several days corresponding to the typical treatment duration of infections. This is achieved with a modular approach; the system integrates a (i) PK module simulating in vivo-like pharmacokinetics by controlling ratios of medium and drug supplying pumps by custom-made programs with (ii) a microfluidic PD device for investigating bacterial cell responses. The PD device features a flat, hydrogel-filled chamber with embedded bacterial cells, mimicking infected tissue, enabling high-resolution time-lapse imaging. Flanking side channels simulate blood vessels by providing a continuous flow of nutrients and antibiotics. The hydrogel confines bacterial movement, with drug and nutrient diffusion mimicking physiological delivery. The system was characterized and validated using reference strains *E. coli* ATCC 25922 and *E. coli* ATCC 35218, as well as an *E. coli* clinical isolate. We investigated the effects of constant and gradient-based drug dosing profiles with different intervals in a comprehensive and systematic experimental series and studied bacterial growth behavior and morphological changes over time, which are not assessable in conventional HFIM.

We administer amoxicillin, a widely used β-lactam antibiotic (time-dependent, bactericidal drug) listed on the WHO’s List of Essential Medicines^37^. Amoxicillin competitively inhibits penicillin-binding proteins (PBPs), blocking glycosyltransferase and transpeptidase activity required for bacterial cell wall cross-linking, which upregulates autolysins and inhibits cell wall synthesis^38^. However, non-dividing cells are tolerant to amoxicillin and can regrow after treatment. Small-scale PK studies have shown amoxicillin’s absorption to be non-linear, which can affect the amount of the drug under different dosing regimens^5^. While the optimal PK/PD index for preventing β-lactam resistance remains unclear^39^, the PK/PD index %fT > MIC is commonly used to guide β-lactam dosing^17,40^. Effective treatment is typically achieved when %fT > MIC is at least 30–50%. If levels fall below the MIC at the infection site, residual pathogens can regrow rapidly^41^. While a high %fT > MIC is related to increased efficacy, an inadequate %fT > MIC is associated with the emergence of resistance and selection of resistant strains. To target strains with β-lactamase-mediated resistance, amoxicillin is frequently combined with clavulanic acid, a β-lactamase inhibitor. β-lactamases are enzymes produced by some bacteria that can hydrolyze β-lactam antibiotics, thereby compromising their effectiveness. Amoxicillin-clavulanic acid shows activity against Gram-negative and -positive bacteria and is used to treat various infections, including respiratory tract infections and urinary tract infections^42^.

## Results

### Design and operation of the microfluidic system (PD module)

**Fig. 1:**
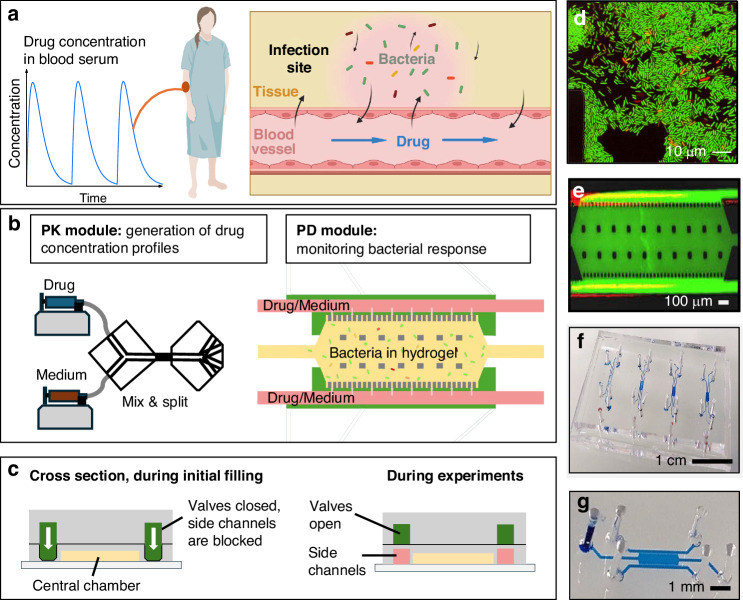
**Microfluidic hollow-fiber infection model (µHFIM) for dynamic antibiotic resistance studies with single-cell resolution.** **a** Schematic of the infection site in the human tissue and drug treatment. **b** The µHFIM consists of two modules: the pharmacokinetic module (PK) supplies the drug profiles of interest, and the pharmacodynamic (PD) module mimics the infection site. **c** Cross sections of the two-layer PD module to illustrate the filling of the central chamber. Bacteria, embedded in hydrogel (yellow), are supplied when the valves (green) are pressurized, thereby blocking the side channels. Media and drugs are applied through the side channels (pink) when the valves are open. Gray color: PDMS, light gray: glass slide. **d** Micrograph of embedded bacteria (merged image: sfGFP producing *E. coli* cells (green), dead cells stained in red (PI). **e** Micrograph of the central cultivation chamber (dimensions: 2.0 mm x 0.6 mm, 1.5 µm high). Parallel pillars confine the central chamber; large pillars inside the chamber prevent the collapse of the ceiling. **f** Photo of four PD modules integrated on a single device. **g** Photo of one PD module

We developed a microfluidic device manufactured in polydimethylsiloxane (PDMS) that represents an infection site in a simplified way (Fig. 1). The infected tissue is mimicked in the flat central chamber by embedding bacteria in a hydrogel (agarose). The “tissue” is defined by an array of pillars and flanked by side channels resembling the blood vessels that transport the drug and medium (Fig. 1a, b). Every device carries four independent, identical chambers to run experiments in parallel (Fig. 1d–g; for detailed information, see Fig. SI 1B, C). The experimental setup starts with filling the central chamber with the bacteria-hydrogel mixture. In contrast to the filling of similar microfluidic devices with large channel heights^43^, special requirements are needed to fill the central chamber with a height of only 1.5 µm. To prevent leakage of the bacterial-hydrogel mixture into the side channels, we blocked the side channels by means of two additional long channels, serving as valves. These are positioned on top of the side channels, separated by a PDMS membrane, and activated during the filling procedure (Fig. 1c). After gel formation, the side channels were opened, and medium or a drug-medium-dye mixture was perfused constantly through these channels, so that nutrients and drugs could diffuse into the hydrogel, and the bacterial cells could be in-line stained.

### Characterization of drug delivery (PK module)

**Fig. 2:**
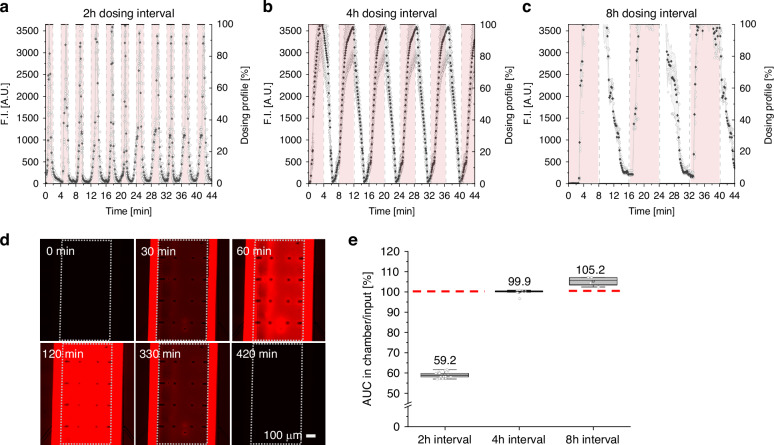
**Characterization of the microfluidic PK module with sulforhodamine B (SRB).** The fluorescence intensity of SRB is measured in the central chamber when supplied in **a** 2-h, **b** 4-h, and **c** 8-h intervals (left axis: determined output profile; right axis: input dosing profile in percent of maximum concentration (100%: 0.1 mM SRB)). **d** Representative fluorescence micrographs of a 4-h interval profile. **e** Determination of area under the curve (AUC) for 2-h peaks (*n* = 17) represents 59.2%, for 4-h peaks (*n* = 8) represents 99.9%, and for 8-h peaks (*n* = 5) represents 105.2% of the input AUC in the output curve

After drug administration, the drug concentration in the human serum is characterized by a quick increase followed by a slight decrease. Here, we create such profiles by using a gradient generator referred to as the PK module (detailed description in Fig. SI 1A and SI Table 1). By varying the flow rates of drug and media, the antibiotic concentration in the microfluidic PD device could be changed over time. It should be noted that the resulting flow rate used here (0.05 µL/min per side channel) is slower than the blood flow rate (in the range of mL/min) but was chosen to prevent rupture of the hydrogel between the supporting posts and reduce the sample consumption to 250 µL/day. We confirmed the profile by monitoring the distribution of a fluorescent dye (sulforhodamine B, SRB) in the central chamber for various administration profiles (Fig. 2a–c, see representative images of one 4 h interval peak Figs. 2d and SI 2A). SRB has a comparable mass to the later used drug amoxicillin (581 vs. 365 Da of amoxicillin and 420 Da in trihydrate form); therefore, we believe that the results can be transferred to amoxicillin and, furthermore, the diffusion is similar to the diffusion in the extracellular matrix of tissue (Fig. SI 3 and considerations in the figure caption). Gradients were formed reproducibly for supply and removal with time delays due to the dead volume of the system (0.98 µL) as well as the effects of diffusion through the gel. The determined area under the curve (AUC) of SRB in the central chamber for 2-h peaks represents the total amount of delivered compound within this cycle and corresponds to 59% of the input. In other words, the c_max_ in the hydrogel shortly reached the c_max_ input concentration. For profiles with 4 h and 8 h intervals, the time was long enough that SRB equilibrated completely (Fig. 2e). The longer intervals are relevant for the standard schedule of amoxicillin administration. If a drug has a shorter administration interval, a narrower chamber will enable the full equilibration within <4 h. The findings of this characterization were later used to calculate the total amount of drug in the central chamber. It should be noted that we could neither observe gradients of SRB along the flow direction of the chamber nor within the hydrogel chamber from one side to the other (Fig. SI 2b, c), and we expect a similar homogeneous distribution for the later supplied drugs. Furthermore, by setting the flow rates for amoxicillin and drug-free medium, the PK module can simulate other pharmacokinetic drug profiles (Fig. SI 4).

### Characterization of cell growth

As cell number and density may influence the results, we next confirmed the reproducibility of inoculation by enumerating the starting number of cells directly after filling (Fig. SI 5A). Cells (sfGFP expressing *E. coli ATCC 25922)* were evenly distributed across different microfluidic chambers with small variations between the days of the experiment (Fig. SI 5B), presumably due to the inaccuracy of the OD_600_ measurement values and cell clumps. This effect is negligible in bulk studies but is visible in the small volume of the microfluidic system. Next, we monitored cell growth and division under constant medium supply by time-lapse fluorescence microscopy. Single-cell resolution was ensured by the low chamber height. The bacteria showed normal growth behavior with a generation time of 32.2 min (Generations (*n*) = 1.875), giving one cell division every 20–30 min (Fig. SI 5D, for representative micrographs over 400 min see Fig. SI 5C) and an exponential growth phase after approximately 2–2.5 h (Fig. SI 6B, for image processing workflow see SI 6A). Thus, the hydrogel restricted the movement of the bacteria but did not compromise cell divisions. Also, the average length of 2.235 µm (Fig. SI 7A, B), and the average cell area of 2.189 µm² (Fig. SI 7C, D) were in the size range expected for normal growing *E. coli* cells. Knowing the average cell area and cell-occupied area, we could derive cell numbers per region of interest and total cell numbers per chamber from the fluorescence images, even without counting the cells (Fig. SI 7E, F).

### Constant drug dosing of amoxicillin

**Fig. 3:**
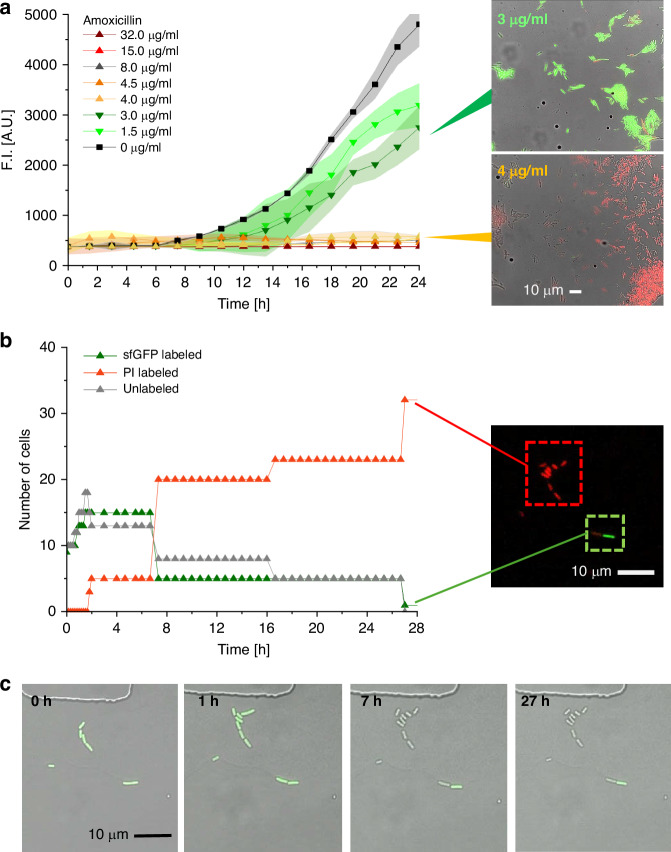
**Constant dosing of amoxicillin on bacterial cells.** **a** MIC determination of *E. coli* ATCC 25922 under constant amoxicillin dosing over 24 h. Growth measured over time as mean fluorescence intensity per POI (*n* = 120 per concentration). **b** Number of sfGFP-containing and PI-labeled cells determined for a representative POI at 4 µg/ml constant dosing and merged, fluorescence microscopy image at *t* = 27 h. **c** Time-lapse microscopy of bacterial cells. Some cells divide at the beginning. In the further time course, all except for one cell lose their sfGFP signal by 27 h

In the next step, we determined the on-chip MIC of the bactericidal antibiotic amoxicillin to the *E. coli* ATCC 25922 cells by conducting time-kill assays at constant drug concentrations between 0 and 32 µg/ml (Fig. 3a, also see growth in % Fig. SI 8). The determined MIC value of 4 μg/ml is in accordance with the MIC given by EUCAST using the static broth microdilution assay^44^. Furthermore, we counted live and dead cells in one smaller area (Fig. 3b). Evaluating the microscopic images (Fig. 3c), we found that dividing cells were killed quickly, in accordance with the mode of action for beta-lactam antibiotics^45^. However, the physiological state of many cells could not be clearly identified as living or dead for several hours. While most of the cells could be identified as dead cells (PI labeled) after 27 h, one cell showed a clear sfGFP signal and no PI signal. Therefore, we assume that the cell is physiologically intact. Previous studies suggested that reduced or stalled growth allows cells to resist antibiotics^46,47^. In contrast to conventional tests, our method facilitates the observation of single-cell heterogeneity, which is crucial for understanding the mode of action of an antibacterial agent on individual cells. We emphasize that these observations reflect single-cell behavior under prolonged antibiotic exposure, while persister cells can be identified only off-chip, which was not done in this study.

### Interval dosing of amoxicillin

**Fig. 4:**
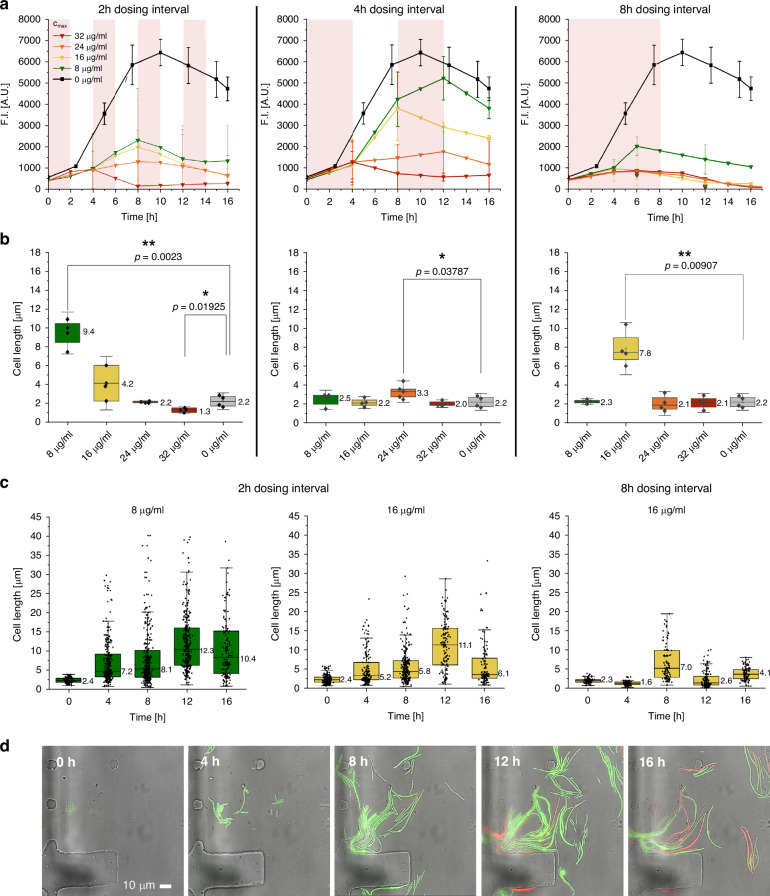
**Gradual dosing of amoxicillin on** ***E. coli*** **ATCC 25922 (sfGFP) cells.** **a** Determination of mean fluorescence intensity of sfGFP over 16 h based on the dosing intervals (2 h, 4 h, 8 h shown from left to right) and antibiotic concentrations (*c*_max_ = 0 µg/ml to 32 µg/ml, *c*_average_ = 50% *c*_max_ [*n* = 240 per concentration and condition]. **b** Distribution of mean bacterial cell length of four treatment conditions (*c*_max_ = 8 µg/ml to 32 µg/ml) compared to the untreated cells for 2h-, 4h- and 8h-dosing interval [*N* = 3 per condition, *n* > 1000, *t*-test α = 0.05] and **c** selected distributions of bacterial cell length based on the dosing interval 2h (*c*_max_ = 8 µg/ml and 16 µg/ml) and 8h (*c*_max_ = 16 µg/ml) over time [*n* > 100 per time point and condition]. **d** Representative micrographs of a time series at 2h-dosing interval (*c*_max_ = 8 µg/ml)

Patients usually take the antibiotic drug repeatedly based on a dosing schedule, which results in serum drug concentrations with intervals of rise and decrease. We employed the PK module to establish such profiles in our system, and investigated systematically different intervals (2 h, 4 h, 8 h in Fig. 4) at four concentrations (*c*_max_ between 8 µg/ml and 32 µg/ml, corresponding to 2× to 8× MIC) (Fig. 4a). In all experiments, *c*_average_ was 50% of *c*_max_ (%fT > MIC of 50%). The PK/PD indices for the experiment were determined over a period of 16 h (Table SI 2). For all dosing profiles, we observed a stop in cell growth within 4 h at the highest administered drug concentration (Fig. 4a). Differences could be found for *c*_average_ of 1× and 2× MIC. Interestingly, 2h- and 8h-dosing intervals inhibited growth more efficiently than the 4h-dosing intervals. Particularly, the efficiency of the short dosing profile was surprising. We assume that the brief recovery period between the doses limited the cells’ ability to adapt to the amoxicillin treatment, since only short subtherapeutic concentrations occur between the doses in short dosing intervals. For the 4h-dosing interval, the biomass at 8 µg/ml and 16 µg/ml showed notable peaks between 4 and 12 h as a sign of sufficient time for recovery and partial regrowth. Around the MIC, the growth/death behavior can differ (see Fig. SI 9 for dosing profiles around the MIC and Table SI 3 for calculated PK/PD indices). The results suggest that the 4h-dosing intervals generate a certain balance between inhibition/killing due to the bactericidal effect of amoxicillin and recovery in the recovery periods between the drug administration, which makes the treatment less effective. As not all bacterial cells were killed in all conditions, the count of sfGFP-labeled cells was still high after 16 h, and these cells may resume growth later, i.e., these cells could be tolerant or persistent.

While bactericidal antibiotics are commonly characterized by how well they prevent bacterial growth or kill bacteria, the effects on cell morphology over time are rarely described. Here, we observed the elongation of bacterial cells for some treatment conditions and analyzed this in more detail (Fig. 4b, c, for all conditions tested over time see Fig. SI 10). Elongated cells were mostly observed at the dosing intervals of 2 h. For this condition, amoxicillin was administered 50% of the time (%fT > MIC = 50%), but only up to a level of approx. 60% of the input level as derived from Fig. 2e, resulting in a *c*_average_ of 4.8 and 2.4 µg/ml, respectively. At the 8-h dosing interval, an elongation of individual cells at c_max_ = 16 µg/ml (*c*_average_ = 8 µg/ml) was observed after 8–10 h of treatment. The average length of the cells increased up to 10 µm—individual filaments could reach a length of up to 40 µm—while the diameter remained unchanged at around 1 µm. Figure 4d shows additionally the temporal development of the cell lengths (more microscopy images in Fig. SI 11). For other dosing profiles with related average drug concentrations, we did not see these elongations. Therefore, not only drug concentration, but also time and duration of drug exposure was a cue for the elongation. Of note, we observed filamentous growth for most of the cells all over the chamber. After the treatment phase, some filamentous growing cells divided into small, normal growing cells, while others died.

### Interval dosing of *E. coli* clinical isolates

**Fig. 5:**
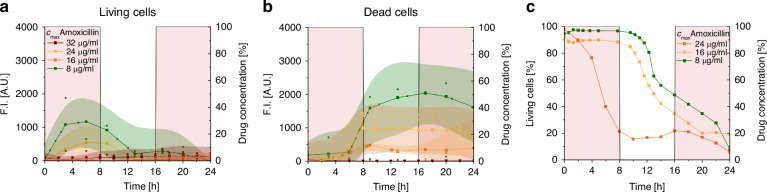
**Treatment of clinical isolates.** **a**–**c** Amoxicillin-sensitive *E. coli* clinical isolate 23060725 behavior over time at different amoxicillin concentrations. Graphs show the signal of live cells (SYTO9-stained) (**a**), and PI-stained dead cells (**b**), as well as the progression of cell viability (% living cells) (**c**) (*N* = 4 chambers per condition; *n* = 320 POI)

Next, we tested a clinical isolate of *E. coli* derived from human blood samples without resistance to amoxicillin. The amoxicillin-sensitive *E. coli* 23060725 clinical isolate was treated with amoxicillin (MIC between 4 and 8 µg/ml, Fig. SI 12). The non-fluorescent clinical isolate could be examined by brightfield microscopy and through on-chip in-line staining with live/dead cell stains (SYTO 9 and PI, respectively). We applied an 8-h dosing interval corresponding to two administrations per day. Figure 5 shows the signals of the live (A) and dead (B) cell stains, from which we could derive the percentage of living cells (C) over time. For the amoxicillin-sensitive isolate, amoxicillin treatment showed a gradual decrease in cell viability for lower concentrations, while the 24 µg/ml amoxicillin treatment caused a sharp drop in cell viability already after 8 h.

### Interval dosing of the drug combination amoxicillin-clavulanic acid

**Fig. 6:**
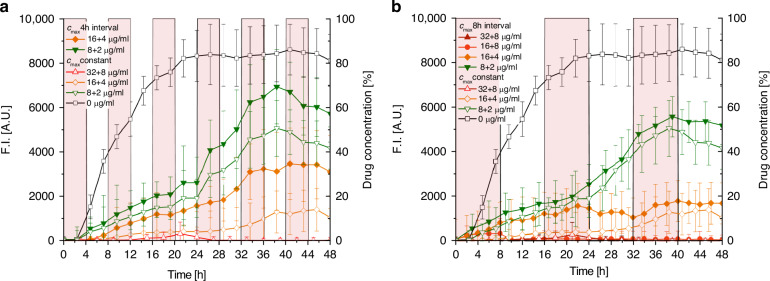
**Dosing of amoxicillin in combination with clavulanic acid [4:1] of amoxicillin-resistant** ***E. coli*** **ATCC 35218 (sfGFP) reference strain.** Gradual dosing in **a** 4-h dosing intervals (8/2 µg/ml, 16/4 µg/ml) and **b** 8-h dosing intervals (8/2 µg/ml, 16/4 µg/ml, 16/8 µg/ml, 32/8 µg/ml) compared with constant dosing (0 µg/ml, 8/2 µg/ml, 16/4 µg/ml, 32 + 8 µg/ml) over 48 h. Growth measured over time as mean fluorescence intensity of the occupied area per POI (*n* = 120 per concentration)

In the clinic, amoxicillin is often administered in combination with clavulanic acid, a ß-lactamase inhibitor, in a ratio of 4:1. Although this has been confirmed as a broadly efficacious combination, high and sustained levels of both compounds are required to successfully treat Gram-negative pathogens, achieved through frequent dosing (three or four times daily)^48^. We therefore extended the use of our µHFIM for studies with this drug combination. We supplied a 4:1 combination of amoxicillin and clavulanic acid on the amoxicillin-resistant *E. coli* ATCC 35218 reference strain, using constant and interval dosing (Fig. 6). Increasing amoxicillin-clavulanic acid concentrations suppressed bacterial growth as expected, with a slightly higher MIC on-chip than in the plate reader (Fig. SI 13). When we compared data with the same absolute amount of drug, e.g., 8/2 µg/ml constant dosing vs. 16/4 µg/ml intervals, the interval dosing was much more efficient. For an 8-h dosing interval, the efficacy of 16/4 µg/ml intervals was almost as good as 16/4 µg/ml constant dosing. Already with 4 µg/ml clavulanic acid, the ß-lactamase activity of the bacteria was strongly inhibited; for the 16/8 µg/ml combination and interval dosing, the growth was completely stopped. For the drug combination amoxicillin-clavulanic acid, these results showed the clear correlation between the concentration of clavulanic acid, the %fT > MIC, and the overall length of the treatment on the effectiveness of the treatment.

## Discussion and conclusion

A key obstacle in understanding bacterial resistance mechanisms is the lack of realistic in vitro systems that adequately represent the complex pharmacodynamics of clinical antibiotic therapies. Our newly developed microfluidic HFIM addresses this gap by combining precisely controllable, time-varying antibiotic concentrations with long-term analysis of bacterial cells. The flat, hydrogel-filled chamber immobilizes bacterial cells and allows for 2D growth, which facilitates the monitoring of cell growth at single-cell resolution. A higher chamber could be used as well, e.g., for studies with a focus on biofilm formation; however, this impedes the option of single-cell enumeration and analysis of filamentous growth. Furthermore, the system allows the continuous observation of bacterial cell reactions under physiologically relevant conditions for at least up to 5 days. In contrast to conventional HFIM systems that consume several litres of medium and drug, and HFIM systems with smaller cartridges that still need about 0.5 L^49^ for operation over several days, our µHFIM has minimal sample requirements (<250 µL/24 h). While we showed here the observation of cell response for regular intervals, the PK module enables the simulation of more complex patterns closely aligned to the real serum concentration in patients, e.g., a fast rise and a slight decrease of the drug concentration during excretion^50^. In summary, our µHFIM provides important methodological advancements over classical PK/PD models.

Our investigations focused on the response of *E. coli* reference strains and clinical isolates to amoxicillin and amoxicillin-clavulanic acid under different exposure regimes, highlighting the differences between constant and dynamic dosing. While the mode of action—the inhibition of peptidoglycan (PG) cross-linking—is well known for amoxicillin^38^, our µHFIM shows comprehensively and systematically the implication of the drug’s pharmacokinetics on the cellular response. Our specific finding for different intervals, with less efficient killing of cells for the 4-h dosing intervals (Fig. 4a), underlines that treatment length and recovery time need careful alignment. Cells can regrow after a single, short pulse above MIC concentrations for 2 or 4 h, respectively. However, recovery of cell division is delayed—possibly due to remaining antibiotic residues that were still bound to negatively charged groups in PG. It is known that PG interacts strongly with positively charged β-lactam antibiotics due to its high density of negatively charged carboxyl and amino groups^51,52^. Since 2 h are not sufficient to remove all bound amoxicillin, short 2-h pulses at high frequency can be as efficient as longer 8-h intervals. In accordance with these results, the dosing profiles of the amoxicillin-resistant reference strains treated with the amoxicillin-clavulanic acid drug combination (Fig. 5) confirm that successful treatment is determined not only by the absolute amount of administered drugs or the proportion of time over MIC (%fT > MIC), but also by the dosing schedule and duration of administration. In other words, lower drug concentrations administered in intervals can be as effective in killing cells as higher concentrations administered continuously. Although these results are specific to amoxicillin, they are likely to be similar for other beta-lactam antibiotics and can be readily investigated in the µHFIM, as well as other antibiotic classes.

One remarkable observation was the pronounced filament formation under amoxicillin exposure. This morphology is often triggered by activation of the SOS response to environmental stress or as a protective mechanism against phagocytosis^53–55^. Elongated cells contain multiple chromosomes and can be 10-50 times longer than typical rod-shaped cells. Filamentation represents a division arrest frequently observed under antibiotic stress^56^. It is known that β-lactam antibiotics induce the formation of filaments both in vitro and in vivo^56–58^, and this phenotype also occurs regularly in clinical samples from antibiotic-treated patients. Subinhibitory concentrations can additionally promote filament formation in *E. coli* and can lead to daughter cells with increased AMR^59^. In our model, this phenotype could not only be mapped with high resolution but also followed in its dynamics over extended periods of time (Fig. 4). This is relevant as filamentous cells are often associated with antibiotic tolerance, intracellular persistence, and treatment failure^59^. Additionally, the decreased surface-to-volume ratio could reduce the influx of antibiotics^60^. However, a comprehensive off-chip analysis would be required to understand the reason and impact of filamentous cell growth on increased tolerance under the conditions that we used here, which is a focus for future work.

These observations would hardly be detectable in classical endpoint analyses and underscore the added value of continuous, high-resolution cell monitoring in our system. Our results demonstrate that morphological plasticity, stress-induced resistance regulation, and cellular stress are integral components of bacterial adaptation to dynamic antibiotic exposure. These often-transient phenotypes are often overlooked since they can hardly be captured with conventional test systems. By combining precise pharmacokinetic control, a tissue-mimicking microenvironment, and long-term single-cell resolution imaging, the µHFIM enables a holistic, time-resolved analysis of bacterial behavior under dynamic antibiotic exposure.

Our findings show that subinhibitory or highly fluctuating drug concentrations, as they occur in human infections, can promote morphological adaptations. This underscores the need to design a treatment regimen based on both MIC values and pharmacokinetic parameters such as dosing intervals and recovery phases. Our results also emphasize that long-term observation of bacterial responses under dynamic antibiotic pressure is essential, as classical endpoint assays often overlook such transient but clinically relevant phenotypes.

Altogether, the µHFIM model provides not only technological innovation but also biological insights into the dynamic interaction between antibiotic action and bacterial adaptation. By bridging dynamic PK control with physiologically relevant microenvironments, it is a versatile tool for in vivo-like investigation of bacterial responses to treatment, allowing visualization and quantification of complex resistance phenotypes. This system may thus support the more efficacious use of available and new antibiotic compounds for bacterial pathogens beyond *E. coli* in the future.

## Materials and methods

A detailed description of materials and cells, device fabrication, characterization, imaging, and data analysis, as well as operation of the device for the various experimental settings, is given in the Supplementary Information.

In short, we used sfGFP-producing *E. coli* reference strains (ATCC 25922 and amoxicillin-resistant ATCC 35218) as well as an amoxicillin-sensitive clinical isolate (*E. coli* 23060725) for our investigations. In a typical experiment, 50 µl of an overnight culture was inoculated in fresh Müller Hinton broth (MHB, Merck) medium and incubated for 4 h. For the experiments, a dilution in fresh MHB media with an OD_600_ of 0.4 was prepared from this culture. 20 µL of the bacterial suspension and 180 µL of 3% fresh prepared agarose hydrogel (Sigma-Aldrich) were mixed and directly filled into the gel inlet of each central chamber. The final bacterial OD_600_ in the hydrogel was 0.04, around 3.2 × 10^7^ cells/ml. After jellification within about 10 min, MHB medium, antibiotic, and dye were supplied in all experiments at a flow rate of 0.05 µl/min per channel with a syringe pump (Nemesys, Cetoni) over the full time of the experiment. Propidium iodide (PI, Sigma-Aldrich) was used as a dead cell stain in all experiments. For the clinical isolates, we permanently supplied 9 µM PI and 2.5 µM life stain SYTO 9 (ThermoFisher Scientific) together with the medium through the side channels. The devices were imaged on a fully automated epifluorescence microscope (Ti2 Eclipse, Nikon). The images were processed and analyzed with custom-made software.

## Supplementary information

Supplementary Information

## Data Availability

Data are available at https://zenodo.org/records/17722905.

## References

[1] World Health Organization (WHO). Surveillance of antimicrobial in Europe, 2022 data—Executive Summary. https://www.ecdc.europa.eu/sites/default/files/documents/Nov2023-ECDC-WHO-executive-Summary.pdf.

[2] Wise, R. et al. Antimicrobial resistance. Is a major threat to public health. *Br. Med. J.* **317**, 609–610 (1998). 9727981 10.1136/bmj.317.7159.609 PMC1113826

[3] Naghavi, M. et al. Global burden of bacterial antimicrobial resistance 1990–2021: a systematic analysis with forecasts to 2050. *Lancet* **404**, 1199–1226 (2024). 39299261 10.1016/S0140-6736(24)01867-1 PMC11718157

[4] Sollier, J. et al. Revitalizing antibiotic discovery and development through in vitro modelling of in-patient conditions. *Nat. Microbiol.* **9**, 1–3 (2024). 38177300 10.1038/s41564-023-01566-w

[5] de Velde, F. et al. Non-linear absorption pharmacokinetics of amoxicillin: Consequences for dosing regimens and clinical breakpoints. *J. Antimicrob. Chemother.* **71**, 2909–2917 (2016). 27330071 10.1093/jac/dkw226

[6] Balaban, N. Q. et al. Definitions and guidelines for research on antibiotic persistence. *Nat. Rev. Microbiol.* **17**, 441–448 (2019). 30980069 10.1038/s41579-019-0196-3 PMC7136161

[7] Artemova, T. et al. Isolated cell behavior drives the evolution of antibiotic resistance. *Mol. Syst. Biol.* **11**, 822 (2015). 26227664 10.15252/msb.20145888 PMC4547850

[8] Clinical and Laboratory Standards Institute (CLSI). *Performance Standards for Antimicrobial Susceptibility Testing* 28th edn. CLSI supplement M100. (Clinical and Laboratory Standards Institute (CLSI), 2018).

[9] Matuschek, E. et al. Development of the EUCAST disk diffusion antimicrobial susceptibility testing method and its implementation in routine microbiology laboratories. *Clin. Microbiol. Infect.* **20**, 255–266 (2014). 10.1111/1469-0691.12373 24131428

[10] Pulido, M. R. et al. Progress on the development of rapid methods for antimicrobial susceptibility testing. *J. Antimicrob. Chemother.* **68**, 2710–2717 (2013). 23818283 10.1093/jac/dkt253

[11] Wiegand, I. et al. Agar and broth dilution methods to determine the minimal inhibitory concentration (MIC) of antimicrobial substances. *Nat. Protoc.* **3**, 163–175 (2008). 18274517 10.1038/nprot.2007.521

[12] Zhang, Q. et al. Acceleration of emergence of bacterial antibiotic resistance in connected microenvironments. *Science* **333**, 1764–1767 (2011). 21940899 10.1126/science.1208747

[13] Baym, M. et al. Multidrug evolutionary strategies to reverse antibiotic resistance. *Science* **351**, 6268 (2016). 10.1126/science.aad3292 PMC5496981 26722002

[14] Toprak, E. et al. Evolutionary paths to antibiotic resistance under dynamically sustained drug selection. *Nat. Genet.* **44**, 101–105 (2012). 10.1038/ng.1034 PMC3534735 22179135

[15] Rosenthal, A. Z. & Elowitz, M. B. Following evolution of bacterial antibiotic resistance in real time. *Nat. Genet.* **44**, 11–13 (2012). 10.1038/ng.1048 22200772

[16] Chevereau, G. et al. Quantifying the Determinants of Evolutionary Dynamics Leading to Drug Resistance. *PLOS Biol.* **13**, e1002299 (2015). 26581035 10.1371/journal.pbio.1002299 PMC4651364

[17] Labreche, M. J. et al. Recent updates on the role of pharmacokinetics-pharmacodynamics in antimicrobial susceptibility testing as applied to clinical practice. *Clin. Infect. Dis.* **61**, 1446–1452 (2015). 26105168 10.1093/cid/civ498

[18] Schaumann, R. et al. Activity of moxifloxacin against Bacteroides fragilis and Escherichia coli in an in vitro pharmacokinetic/pharmacodynamic model employing pure and mixed cultures. *J. Med. Microbiol.* **54**, 749–753 (2005). 16014428 10.1099/jmm.0.45994-0

[19] Velkov, T. et al. PK/PD models in antibacterial development. *Curr. Opin. Microbiol.* **16**, 573–579 (2013). 23871724 10.1016/j.mib.2013.06.010 PMC3834155

[20] Cadwell, J. J. S. The hollow fiber infection model: principles and practice. *Adv. Antibiot. Antibodies* **1**, 101 (2015).

[21] Cadwell, J. J. S. The hollow fiber infection model for antimicrobial pharmacodynamics and pharmacokinetics. *Adv. Pharmacoepidemiol. Drug Saf.* **S1**, 007 (2012).

[22] Gumbo, T. et al. Nonclinical models for antituberculosis drug development: a landscape analysis. *J. Infect. Dis.* **211**, 83–95 (2015). 10.1093/infdis/jiv183 26009617

[23] Dewachter, L. et al. Bacterial heterogeneity and antibiotic survival: understanding and combatting persistence and heteroresistance. *Mol. Cell* **76**, 255–267 (2019). 31626749 10.1016/j.molcel.2019.09.028

[24] Hol, F. J. H. & Dekker, C. Zooming in to see the bigger picture: microfluidic and nanofabrication tools to study bacteria. *Science* **346**, 1251821 (2014). 25342809 10.1126/science.1251821

[25] Said, B. S. & Or, D. Synthetic microbial ecology: engineering habitats for modular consortia. *Front. Microbiol.* **8**, 1125 (2017). 28670307 10.3389/fmicb.2017.01125 PMC5472676

[26] Kim, H. J. et al. Human gut-on-a-chip inhabited by microbial flora that experiences intestinal peristalsis-like motions and flow. *Lab Chip* **12**, 2165–2174 (2012). 22434367 10.1039/c2lc40074j

[27] Li, B. et al. Gradient microfluidics enables rapid bacterial growth inhibition testing. *Anal. Chem.* **86**, 3131–3137 (2014). 24548044 10.1021/ac5001306 PMC3988682

[28] Kalinin, Y. et al. Responses of *Escherichia coli* bacteria to two opposing chemoattractant gradients depend on the chemoreceptor ratio. *J. Bacteriol.* **192**, 1796–1800 (2010). 20118262 10.1128/JB.01507-09 PMC2838042

[29] Haessler, U. et al. An agarose-based microfluidic platform with a gradient buffer for 3D chemotaxis studies. *Biomed. Microdevices* **11**, 827–835 (2009). 19343497 10.1007/s10544-009-9299-3

[30] Grünberger, A. et al. Spatiotemporal microbial single-cell analysis using a high-throughput microfluidics cultivation platform. *Cytom. Part A.* **87A**, 1101–1115 (2015). 10.1002/cyto.a.22779 26348020

[31] Wang, P. et al. Robust growth of Escherichia coli. *Curr. Biol.* **20**, 1099–1103 (2010). 20537537 10.1016/j.cub.2010.04.045 PMC2902570

[32] Bamford, R. A. et al. Investigating the physiology of viable but non-culturable bacteria by microfluidics and time-lapse microscopy. *BMC Biol.* **15**, 121 (2017). 29262826 10.1186/s12915-017-0465-4 PMC5738893

[33] Song, J. et al. A microfluidic device for studying chemotaxis mechanism of bacterial cancer targeting. *Sci. Rep.* **8**, 6394 (2018). 29686328 10.1038/s41598-018-24748-7 PMC5913277

[34] Choi, J. et al. Rapid antibiotic susceptibility testing by tracking single cell growth in a microfluidic agarose channel system. *Lab Chip* **13**, 280–287 (2013). 23172338 10.1039/c2lc41055a

[35] Kim, K. et al. Visual estimation of bacterial growth level in microfluidic culture systems. *Sensors* **18**, 447 (2018). 29401651 10.3390/s18020447 PMC5855051

[36] Kim, S. et al. Microfluidic-based observation of local bacterial density under antimicrobial concentration gradient for rapid antibiotic susceptibility testing. *Biomicrofluidics* **13**, 014108 (2019). 30867878 10.1063/1.5066558 PMC6404913

[37] World Health Organization (WHO). Model List of Essential Medicines—23rd List, 2023. *The Selection and Use of Essential Medicines 2023*. https://www.who.int/publications/i/item/WHO-MHP-HPS-EML-2023.02.

[38] Sauvage, E. & Terrak, M. Glycosyltransferases and transpeptidases/penicillin-binding proteins: valuable targets for new antibacterials. *Antibiotics* **5**, 12 (2016). 27025527 10.3390/antibiotics5010012 PMC4810414

[39] Stearne, L. E. T. et al. Effect of dosing and dosing frequency on the efficacy of ceftizoxime and the emergence of ceftizoxime resistance during the early development of murine abscesses caused by *Bacteroides fragilis* and *Enterobacter cloacae* mixed infection. *Antimicrob. Agents Chemother.* **51**, 3605–3611 (2007). 17646416 10.1128/AAC.01486-06 PMC2043274

[40] Martinez, M. N. et al. Dosing regimen matters: the importance of early intervention and rapid attainment of the pharmacokinetic/pharmacodynamic target. *Antimicrob. Agents Chemother.* **56**, 2795–2805 (2012). 22371890 10.1128/AAC.05360-11 PMC3370717

[41] Póvoa, P. et al. Optimizing antimicrobial drug dosing in critically ill patients. *Microorganisms* **9**, 1401 (2021). 34203510 10.3390/microorganisms9071401 PMC8305961

[42] Delgado-Valverde, M. et al. MIC of amoxicillin/clavulanate according to CLSI and EUCAST: discrepancies and clinical impact in patients with bloodstream infections due to Enterobacteriaceae. *J. Antimicrob. Chemother.* **72**, 1478–1487 (2017). 28093484 10.1093/jac/dkw562

[43] Hirth, E. et al. Self-assembled and perfusable microvasculature-on-chip for modeling leukocyte trafficking. *Lab Chip* **24**, 292–304 (2023). 10.1039/d3lc00719g PMC10793075 38086670

[44] European Committee on Antimicrobial Susceptibility Testing (EUCAST). Routine and Extended Internal Quality control for MIC Determination and Disk diffusion as Recommended by EUCAST; Version 15.0. https://aurosan.de/images/mediathek/servicematerial/EUCAST_RefStaemme_Sollwerte.pdf (2025).

[45] Tuomanen, E. et al. The rate of killing of Escherichia coli by ß-lactam antibiotics is strictly proportional to the rate of bacterial growth. *J. Gen. Microbiol.* **132**, 1297–1298 (1986). 3534137 10.1099/00221287-132-5-1297

[46] Brauner, A. et al. Distinguishing between resistance, tolerance and persistence to antibiotic treatment. *Nat. Rev. Microbiol.* **14**, 320–330 (2016). 27080241 10.1038/nrmicro.2016.34

[47] Steel, H. & Papachristodoulou, A. The effect of spatiotemporal antibiotic inhomogeneities on the evolution of resistance. *J. Theor. Biol.* **486**, 110077 (2020). 31715181 10.1016/j.jtbi.2019.110077

[48] Huttner, A. et al. Oral amoxicillin and amoxicillin–clavulanic acid: properties, indications and usage. *Clin. Microbiol. Infect.* **26**, 871–879 (2020). 31811919 10.1016/j.cmi.2019.11.028

[49] Hayashi, Y. et al. Establishment and validation of downsized hollow-fibre infection model and pharmacokinetics/pharmacodynamics analysis of VAN on Enterococcus faecium. *J. Antimicrob. Chemother.* **80**, 2092–2099 (2025). 40437892 10.1093/jac/dkaf175 PMC12313456

[50] Sun, P. et al. The bioavailability and pharmacokinetics of an amoxicillin–clavulanic acid granular combination after intravenous and oral administration in swine. *J. Vet. Pharmacol. Ther.* **44**, 126–130 (2021). 33063331 10.1111/jvp.12916

[51] Vollmer, W. et al. Peptidoglycan structure and architecture. *FEMS Microbiol. Rev.* **32**, 149–167 (2008). 18194336 10.1111/j.1574-6976.2007.00094.x

[52] Malanovic, N. & Lohner, K. Antimicrobial peptides targeting Gram-positive bacteria. *Pharmaceuticals* **9**, 59 (2016). 27657092 10.3390/ph9030059 PMC5039512

[53] Zhang, D. et al. Molecular responses during bacterial filamentation reveal inhibition methods of drug-resistant bacteria. *Proc. Natl. Acad. Sci. USA* **120**, e2301170120 (2023). 37364094 10.1073/pnas.2301170120 PMC10318954

[54] Khan, F. et al. Filamentous morphology of bacterial pathogens: regulatory factors and control strategies. *Appl. Microbiol. Biotechnol.* **106**, 5835–5862 (2022). 35989330 10.1007/s00253-022-12128-1

[55] Yang, D. C. et al. Staying in shape: the impact of cell shape on bacterial survival in diverse environments. *Microbiol. Mol. Biol. Rev.* **80**, 187–203 (2016). 26864431 10.1128/MMBR.00031-15 PMC4771367

[56] Justice, S. S. et al. Morphological plasticity as a bacterial survival strategy. *Nat. Rev. Microbiol.* **6**, 587–594 (2008). 10.1038/nrmicro1820 18157153

[57] Hendri, N. A. M. et al. Ultrastructural and morphological studies on variables affecting Escherichia coli with selected commercial antibiotics. *Cell Surf.* **11**, 100120 (2024). 38313869 10.1016/j.tcsw.2024.100120 PMC10831149

[58] Yao, Z. et al. Distinct single-cell morphological dynamics under beta-lactam antibiotics. *Mol. Cell* **48**, 705–712 (2012). 23103254 10.1016/j.molcel.2012.09.016 PMC3525771

[59] Bos, J. et al. Emergence of antibiotic resistance from multinucleated bacterial filaments. *Proc. Natl. Acad. Sci. USA* **112**, 178–183 (2015). 25492931 10.1073/pnas.1420702111 PMC4291622

[60] Ojkic, N. et al. Antibiotic resistance via bacterial cell shape-shifting. *mBio.* **13**, e0065922 (2022). 35616332 10.1128/mbio.00659-22 PMC9239207

