# Supplementary material for: A microfluidic hollow-fiber infection model (µHFIM): monitoring bacterial response to dynamic drug treatment with single-cell resolution
Source: Microsyst Nanoeng. 2026 Jul 9;12:259. doi: 10.1038/s41378-026-01377-z (PMC13351019; doi:10.1038/s41378-026-01377-z)
Supplement: Supplementary file 1 — Supplementary Information [file 41378_2026_1377_MOESM1_ESM.pdf]

Supplementary Information

A microfluidic hollow-fiber infection model (µHFIM): monitoring bacterial response to dynamic drug treatment with single-cell resolution

Friederike-Leonie Born[1], Raphael Dezauzier[1], Annelies S. Zinkernagel[2], and Petra S. Dittrich[1*]

[1] Department of Biosystems Science and Engineering, ETH Zurich, Basel, Switzerland
[2] Department of Infectious Diseases and Hospital Epidemiology, University Hospital Zurich, University of Zurich, Zurich, Switzerland

***Corresponding author:**
Petra S. Dittrich
ETH Zürich, Department Biosystems Science and Engineering
Schanzenstrasse 44
CH-4056 Basel, Switzerland

Phone: +41 61 387 33 10
Email: petra.dittrich@bsse.ethz.ch
ORCID: 0000-0001-5359-8403

Content

1. Materials and Methods
    1.1. Design and fabrication of the µHFIM platform
    1.2. Drug solutions
    1.3. Bacterial cultivations
    1.4. Device preparation and operation
    1.5. Imaging of the microfluidic device
    1.6. Data analysis
    1.7. Validation of the in-vitro model using reference *E. coli* strains
    1.8. PK/PD studies
    1.9. Antibiotic treatment on *E. coli* clinical isolates

2. Figures 1 -13 and Tables 1-3

3. References

1. Materials and Methods

1.1. Design and fabrication of the microfluidic µHFIM platform

1.1.1. Microfluidic device fabrication (PD device)

We used a two-layer polydimethylsiloxane (PDMS) microfluidic device. The bottom layer contained a central cultivation chamber (2000 µm x 600 µm, 1.5 µm high, chamber volume 2.139 x 10 $^{-6}$ ml) and fluidic side channels (100 µm width, 10 µm high) on both sides, separated by an array of pillars. The top layer (pressure layer) of the device contained only the side channels with a height of 20 µm to block the channels underneath, when the central chamber is filled with hydrogel. The microfluidic device is manufactured according to previous protocols (double-layer device [1]). Briefly, the designs of the top and bottom layer were drawn with AutoCAD (Autodesk GmbH) and printed by Selba S.A (Switzerland). This design was transferred on two 4-inch silicon wafers, which were coated with negative photoresist SU8 to a height of 1.5 µm or 20 µm (SU8 6001 and SU8 3010 from micro resist technology). After spin coating the SU8 a pre-exposure bake, UV light exposure (MA-7 mask aligner, Suss Microtec) and post-exposure bake was carried out (bottom layer: SU8 6001, speed: 1500 rpm, bake: 1 min at 65°C, 3 min at 95°C, exposure dose: 400 mJ cm^{-2}, post exposure bake: 1 min at 65°C, 2 min at 95°C; top layer: SU8 3010, speed: 3000 rpm, bake: 1 min at 65°, 3 min at 95°, exposure dose: 600 mJ cm^{-2}, post exposure bake: 1 min at 65°C and 2 min at 95°C). SU8 was developed in an mr-Dev 600 developer (Micro Resist Technology GmbH). The final master mold wafers were hard baked (2 hours ramp from 25°C to 200°C, stay at 200°C for 2 h, cool down) and silanized by perfluorooctyl trichlorosilane (PFOTS, Merck). The microfluidic devices were then fabricated from PDMS (Sylgard 184, Dow Corning) using soft lithography techniques. For the bottom layer, we used 5 g PDMS with a ratio of 1:8 of curing agent to polymer, for the top layer we used 40 g PDMS with a ratio of 1:10. The mixture for the bottom layer was spin-coated on top of the Si wafer to a height of ∼25 µm (20 s at 500 rpm and 1 min at 2500 rpm) and cured for 2 h at 80 °C. The mixture for the top layer was poured over the other Si wafer to obtain a height of 3 mm, and cured overnight at 80 °C. The top layer was cut into individual devices, and the inlet and outlet holes were manually punched with a 1 mm biopsy puncher. The cured layers were then aligned under a microscope and bonded together with an intermediate layer of spin-coated curing agent, removed from the wafer, cut into pieces and holes were punched through the bottom layer. In the next step, the PDMS device was bonded to a glass slide (no. 1: 0.13 - 0.17 mm thick), that was cleaned and sterilized with 70% ethyl alcohol. The PDMS device and the glass slide were exposed to an oxygen plasma (Harrick Plasma Cleaner PDC-32G) for 45 seconds to

activate the surfaces. Afterward, the PDMS was gently pressed to the glass slide to obtain a stable bonding.

1.1.2 Modular gradient generator (PK Module)

The modular gradient generator consists of syringe pumps (Nemesys, Cetoni) with 250 µl or 1000 µl glass syringes (Agilent), a merging chip (up to 4 inlets, one outlet) and a splitting chip (one inlet and up to 4 outlets) made of PC. The channels in these modules have an inner diameter of 100 µm. Additionally, we use capillary tubing (PEEK) with an inner diameter of 50 µm and Luer adapters (PEEK) for connecting capillaries and modules. The system has an internal volume of less than 10 µl. This modular approach leads to a high degree of flexibility in carrying out experiments. When delivering a single drug, two input channels (one for drug and one for media) were used. The flow rates between the individual pumps were varied over time to deliver the desired profile to two chambers. Thereby, the total flow rate of all pumps was kept constant in all experiments at 0.2 µl/min to achieve a maximum flow rate per channel of 0.05 µl/min.

1.1.3 Characterization of microfluidic system

To visualize delivery and diffusion of the drug in the central chamber, a 0.1 mM solution of fluorescent sulforhodamine B was used as "drug model", and was supplied in dosing intervals of 2h, 4h and 8h. The input profiles were defined by the pumping ratios, and the output profiles were determined by the mean fluorescence intensity in the central chamber. Finally, the area under the curve (AUC) was calculated according to the simps method in the Python package scipy.integrate, from which the differences in input and output profiles were derived. In addition, the flow behavior was simulated using COMSOL (COMSOL Inc) Version Nr. 6.1.

1.2 Drug solutions

Stock solutions of amoxicillin (Sigma-Aldrich) and clavulanic acid (Sigma-Aldrich) were prepared with a concentration of 2 mg/ml in phosphate buffer (pH 6). In addition, stock solutions of the premixed amoxicillin-clavulanic acid (4:1) (Sigma-Aldrich) with a concentration of 2 mg/ml amoxicillin and 0.5 mg/ml clavulanic acid were prepared. Aliquots with 100 µl of all stock solutions were stored at – 20 °C and thawed only once, without refreezing. Solutions of antibiotics in media were prepared shortly before the experiments from these aliquots. Kanamycin sulfate with 100x concentration (Sigma-Aldrich) for plasmid maintenance was stored in 0.5 mL aliquots (-20 °C).

1.3 Bacterial cultivation

We used *Escherichia coli* (Migula) Castellani and Chalmers ATCC 25922 and *E. coli* (Migula) Castellani and Chalmers ATCC 35218 (amoxicillin resistance) reference strains (ATCC collection). The strains were modified with pSEVA271-sfgfp plasmid with kanamycin resistance to overexpress the gene for the superfolder green fluorescent protein (sfGFP) (lab collection Bioprocess Laboratory, ETH Zurich generated by Steven Schmitt[2]). Cryo-stocks of the bacterial strains were stored at -80 °C. The inoculation was done with an inoculation loop from the cryo-stock into 3 ml MHB, cation-adjusted, containing appropriate antibiotics for plasmid maintenance (for *E. coli* ATCC 25922: 50 µg/ml kanamycin sulfate and for *E. coli* ATCC 35218: 50 µg/ml kanamycin sulfate and 50 µg/ml amoxicillin). The liquid culture was grown at 37 °C by using a shaking incubator (Minitron, Infors HT) with a shaking speed of 220 rpm. A 50 µl portion of the culture was transferred into fresh MHB media and incubated overnight. Subsequently, 50 µl were inoculated in fresh MHB media and incubated for 4 hours. For the experiments a dilution in fresh MHB media with OD_{600} of 0.4 was prepared from this culture (final dilution in hydrogel OD_{600} = 0.04, around 3.2 x 10^7 cells/ml). *E. coli* ATCC 35218 bacterial cells were washed two times with media without amoxicillin before the experiments with amoxicillin - clavulanic acid. In addition, a clinical isolate of *E. coli* (lab collection Prof. Annelies S. Zinkernagel and Prof. Silvio D. Brugger, Department of Infectious Diseases and Hospital Epidemiology, University of Zurich, Zurich) was used, stored and cultivated in MHB under BSL2 conditions.

1.4 Device preparation and operation

We used 3% LM agarose (Sigma-Aldrich) hydrogel in all experiments. The agarose solution was heated up to 95 °C and then kept at 45 °C until use. An empty microfluidic device with four chambers was placed in a customized metal holder on the microscopy stage. The environmental box of the microscope was pre-heated to 37 °C and maintained at this temperature during the whole on-chip experiment. The top (pressure) layer was filled with water and was pressurized (1.8 bar) to close the side channels and pillar array. The gel was cooled down to a temperature of 35-40 °C, and 180 µl 3% LM Agarose and 20 µl bacterial culture were mixed. This mixture was directly pipetted into the gel inlet of each central chamber (final bacterial OD_{600} in the gel 0.04). Formation of the hydrogel occurred under these conditions within 10 min. The status of the gel formation was assessed by the bacterial movement. After gel formation, the top layer was deactivated (0 bar) to open the side channels for the following experiment. MHB medium, antibiotic and dye were supplied in all experiments at a flow rate of 0.05 µl/min per channel with a syringe pump (Nemesys, Cetoni) over the full time of the experiment. Propidium iodide (PI) (Sigma Aldrich, 1.5 mM stock solution) was used as dead cell stain in all experiments.

1.5 Imaging of the microfluidic device

For time-lapse imaging, we used a fully automated epifluorescence microscope (Ti2 Eclipse, Nikon) controlled with NIS-Elements Advanced Research software (Nikon) with a DS-Qi2 CMOS camera (Nikon) and a SOLA II LED light source (Lumencor). We selected imaging regions (points of interests, POI) all over the central chambers of each device and imaged up to 120 h in brightfield and epifluorescence mode. Imaging was performed by using a 100× objective (100x Apochromat TIRF oil objective, Nikon). For epifluorescence microscopy, the following parameters and optical filters were used: sfGFP and SYTO9 fluorescence was measured with the 30 % LED intensity, 470/40 excitation filter, 495 dichroic, 525/50 emission filter, exposure time 100 ms. SRB fluorescence was measured with a 535/40 nm excitation filter, a 565 nm dichroic mirror, a 590/40 barrier filter, and an exposure time of 50 ms at 30% LED intensity. Propidium iodide (PI) was measured with a 570/40 nm excitation filter, a 600 nm dichroic mirror, a 645/75 barrier filter, and an exposure time of 100 ms at 30% LED intensity.

1.6 Data analysis

The experiments with fluorophore gradients were analysed with customized Matlab Software (MathWorks) developed in our lab. The analysis of the occupied area (biomass) and fluorescence intensity of the occupied area were carried out by a customized program developed in our lab using Python (Python Software Foundation, version 3.12). The growth rate was determined by image analysis based on fluorescence measurements of sfGFP of the area occupied by cells per POI. The area occupied by the cells per POI was measured by a customized Python program, which includes thresholding and otsu filtering. The method to determine growth by means of the increase of biomass was also described by Choi et al [3]. The mean fluorescence intensity per time point was then determined from this occupied area. Bacteria counting and the analysis of the cell length and area, as well as the analysis of the length of inclusion bodies were done using NIS Elements (Nikon Instruments Inc). All graphs were produced using OriginPro (version 2024/2025, OriginLab Corporation).

1.7 Validation of the in-vitro model using reference *E. coli* strains

1.7.1 Determination of the starting cell number and bacterial growth on-chip

The inoculum of the *E. coli* ATCC 25922 pSEVA271-sfgfp strain was prepared and the hydrogel with bacterial cells was filled into the device. The variations in the initial bacterial cell count (N_0) from device to device, from chamber to chamber and from experiment to experiment were analysed with the software NIS Elements. This was done in two devices of 2 consecutive experimental days (N=5

chambers, n=347 POI). In addition, we compared the mean initial cell number of the 4 chambers per device from 14 independent experiments (N=14, n=56 chambers).

During the experiments at 37 °C, MHB media was supplied constantly for 16 h, while imaging was performed every 2 h. The growth rate was determined by image analysis based on fluorescence measurements of sfGFP of the area occupied by cells per POI (N=4 chambers, n=366 POI). Cell length (n=2706) and cell area (n=500) of the bacteria under growth condition were determined manually using NIS elements. We used the data of the occupied area and the average area per cell to estimate the number of cells per POI and per chamber.

1.7.2 Determination of MIC under constant amoxicillin dosing and carrying out time-kill assays

The MIC of amoxicillin for the tested *E. coli* ATCC 25922 pSEVA271-sfgfp strain was determined on-chip using constant drug dosing (Note: this is not the static MIC as in standard assays, since the concentration was constantly delivered over the full time of experiment) and time-lapse microscopy over 24 h. The antibiotic solutions were supplied at a constant flow rate for 24 h at 37 °C. Nine amoxicillin concentrations between 0 µl/ml and 32 µg/ml were tested including 4 µg/ml, which is the static MIC for amoxicillin determined by CLSI[4]. The MIC and growth/kill behaviour were determined by image analysis based on fluorescence measurements of sfGFP and PI (3.0 µM). In addition, the behaviour of cells in individual POIs were investigated at 4 µl/ml and the growth/death curves of the single cells in the POI were determined.

1.8 PK/PD studies

1.8.1 Amoxicillin gradients with 2h-, 4h- and 8h- dosing intervals tested on an amoxicillin sensitive *E. coli* ATCC 25922 strain

In the next step, the effect of three dosing intervals (2h, 4h and 8h) was tested over a period of 16h at 37 °C on *E. coli* ATCC 25922 pSEVA271-sfgfp (with sfGFP expression). Four concentration gradients were tested for all three dosing intervals, ranging from c_{min}= 0 µl/min to c_{max} between 8 µg/ml and 32 µg/ml. Average concentrations ($c_{average}$), defined as 50 % of c_{max}, were between 4 µg/ml to 16 µg/ml (1xMIC to 4xMIC). For time-lapse microscopy with 100x objective, each chamber was divided into 60 POIs. Per concentration and condition n = 240 POI were analysed. For the tested dosing intervals around the MIC the gradient was defined by the mean concentration of 4 µl/ml. The c_{min} and c_{max} were varied in the intervals in five ranges between 0 µg/ml and 8 µg/ml and compared to constant 4 µg/ml (see figure S8). Growth and death behaviour were determined by image analysis based on fluorescence measurements of sfGFP and PI (3.0 µM) of the area occupied by cells per POI. Further PK/PD parameters were investigated, e.g. the area under the time-concentration curve (AUC), the ratio between the maximum concentration and the MIC (c_{max}/MIC) and the time during which the concentration exceeds the MIC (T > MIC). For calculating the expected area under the curve of the output profiles (fAUC) the trapezoidal rule was used and based on the determined profiles with SRB, the real output fAUC was calculated. The introduced amount of antibiotic equals the unbound fraction of the drug, since the protein-bound fraction could be neglected in the platform. In addition, morphological changes in the bacterial shape caused by the drug treatment were investigated. In this context, the change in bacterial length between the different concentrations and dosing intervals were analysed using NIS elements (Nikon). For the statistical analysis a t-test was used (α=0.05; N=3 per condition n>1000 cells analysed). All POI per chamber were considered as one experiment. After testing for normal distribution, the mean per chamber of all experiments per condition were compared with the standard group (growth without treatment).

1.8.2 Amoxicillin-clavulanic acid combinatory treatment tested on amoxicillin resistant *E. coli* ATCC 35218

An amoxicillin resistant *E. coli* ATCC 35218 reference strain was used for investigating the treatment effects of 4h- and 8h- dosing interval combinatory treatment of amoxicillin-clavulanic acid [4:1]

compared to constant dosing. The strain was used with a pSEVA271-sfgfp plasmid to facilitate the imaging analysis. Due to the plasmid-based resistance, it was necessary to continuously culture the strain with amoxicillin in MHB media to maintain resistance. During the experiments no additional amoxicillin was used beside the amount of amoxicillin–clavulanic acid given. Therefore, the culture was washed with media containing no amoxicillin, before diluting it to the desired OD. For constant dosing four concentrations between 0 µg/ml (NK) and 32+8 µg/ml amoxicillin-clavulanic acid (%T>MIC=100 %) were tested. For the 4h dosing interval dosing profiles 8/2 µg/ml and 16/4 µg/ml and for the 8h dosing interval dosing profiles four concentrations between 8/2 µg/ml and 32/8 µg/ml were investigated over 48 hours (%T >MIC=50 %).

1.9 Antibiotic treatment on *E. coli* clinical isolate

An amoxicillin sensitive (*E. coli* 23060725) *E. coli* clinical isolate (from blood) was tested. The combination of SYTO 9 (excitation/emission maxima ~480/500 nm) and PI (excitation/emission maxima ~490/635 nm) was used to differentiate between live and dead cells. PI penetrates only cells with disrupted membranes and is generally excluded from viable cells. In contrast, the green-fluorescent nucleic acid stain SYTO9 enters live and dead bacterial cells [5,6]. Life stain SYTO 9 (2.5 µM) and dead stain propidium iodide (PI) (9.0 µM) were used according to the optimized concentration protocol by Ou et al (2016)[7]. A 5 µM stock solution of SYTO 9 in DMSO was prepared and stored at - 20 °C. The unlabelled, clinical isolate was adjusted to an OD_{600} of 0.4 and then pre-incubated with the life and dead stains for 30 min on ice before injection with hydrogel into the device. The strains were imaged every 2 h up to several days. During the entire test period, life and death stain was applied with the medium and antibiotic through the side channels so that the cells were permanently stained green and red, respectively. Growth/death behaviours were determined via image analysis based on fluorescence measurements of the life/death cell staining reagents and the percentage of living cells was calculated based on the ratio of PI/SYTO 9 [7]:

$$\%\ live\ cells\ \propto (100 * \left(\frac{SYTO\ 9}{PI}\right))/(1 + \left(\frac{SYTO\ 9}{PI}\right))$$

2. Figures and Tables

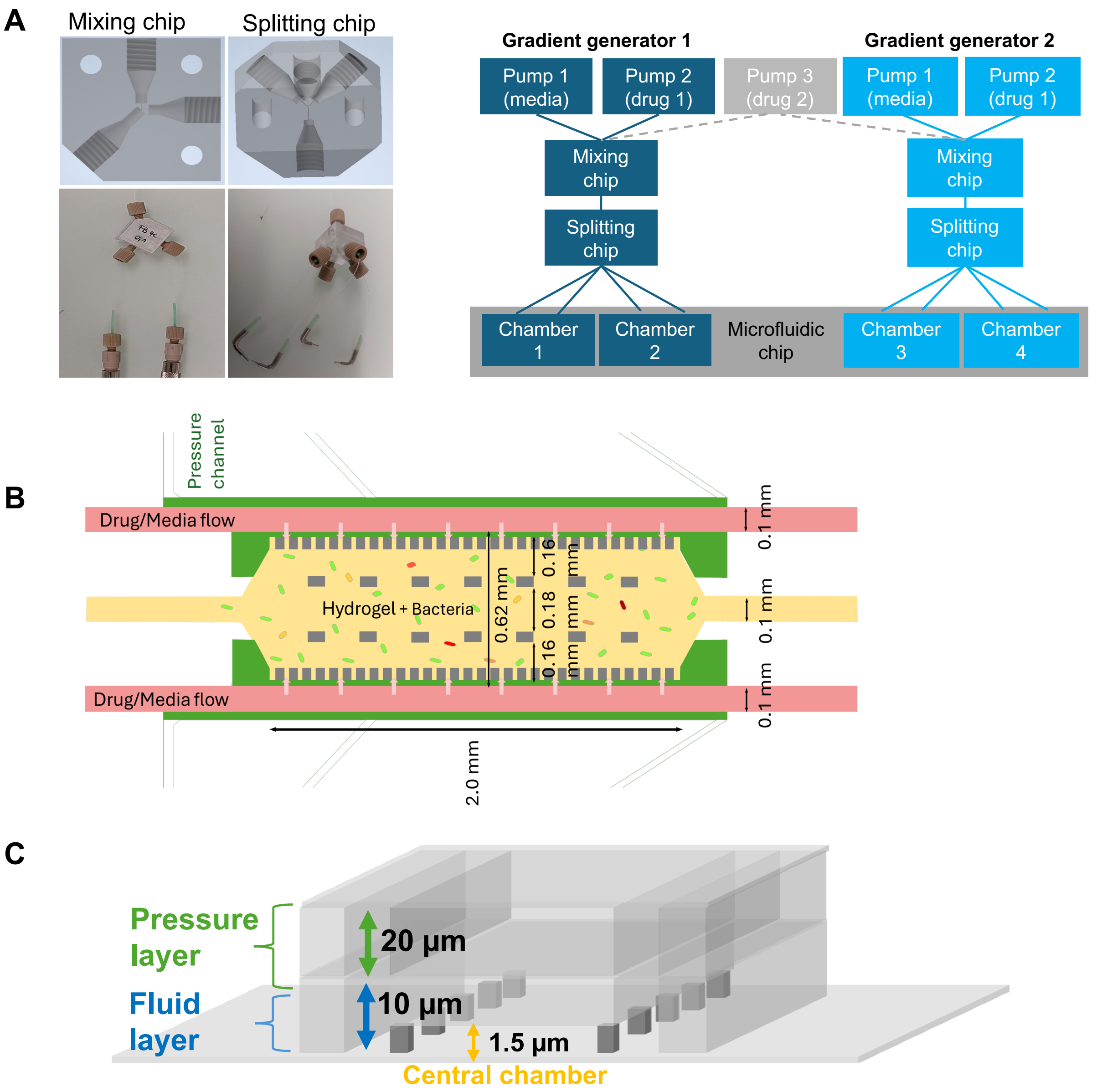

Figure SI 1. *Microfluidic platform for dynamic antibiotic resistance studies with single-cell resolution. The assembled µHFIM consists of a modular PK module for gradient generation, and the two-layer microfluidic PD device where bacterial cultivation, drug studies and imaging take place. (A) Gradient generation (PK) module consisting of a 3D-printed "mixing" chip, that is connected via tubing with a 3D-printed splitting chip with four outlets. Top: technical drawings and photographs of the chips; bottom: schematics of the connections with lines representing tubing. (B,C) Drawings of the PD module with detailed dimensions and heights.*

Table SI 1: *Detailed dimensions of the PK module and total dead volume.*

Element	Inner diameter	length	Inner volume	Flow velocity	Flow regime
**tubing**	50 µm	10 cm	0.2 µl	1.7 mm/s	Laminar flow (Re =0.12)
**mixing chip**	100 µm	3.5 mm	0.03 µl	0.19 mm/s	Laminar flow (Re =0.04)
**tubing**	50 µm	30 cm	0.59 µl	1.7 mm/s	Laminar flow (Re =0.12)
**splitting chip**	150 µm	9.2 mm	0.16 µl	0.42 mm/s	Laminar flow (Re =0.06)
**tubing**	50 µm	10 cm	0.2 µl	1.7 mm/s	Laminar flow (Re =0.12)
**Total dead volume**	1.18 µl				

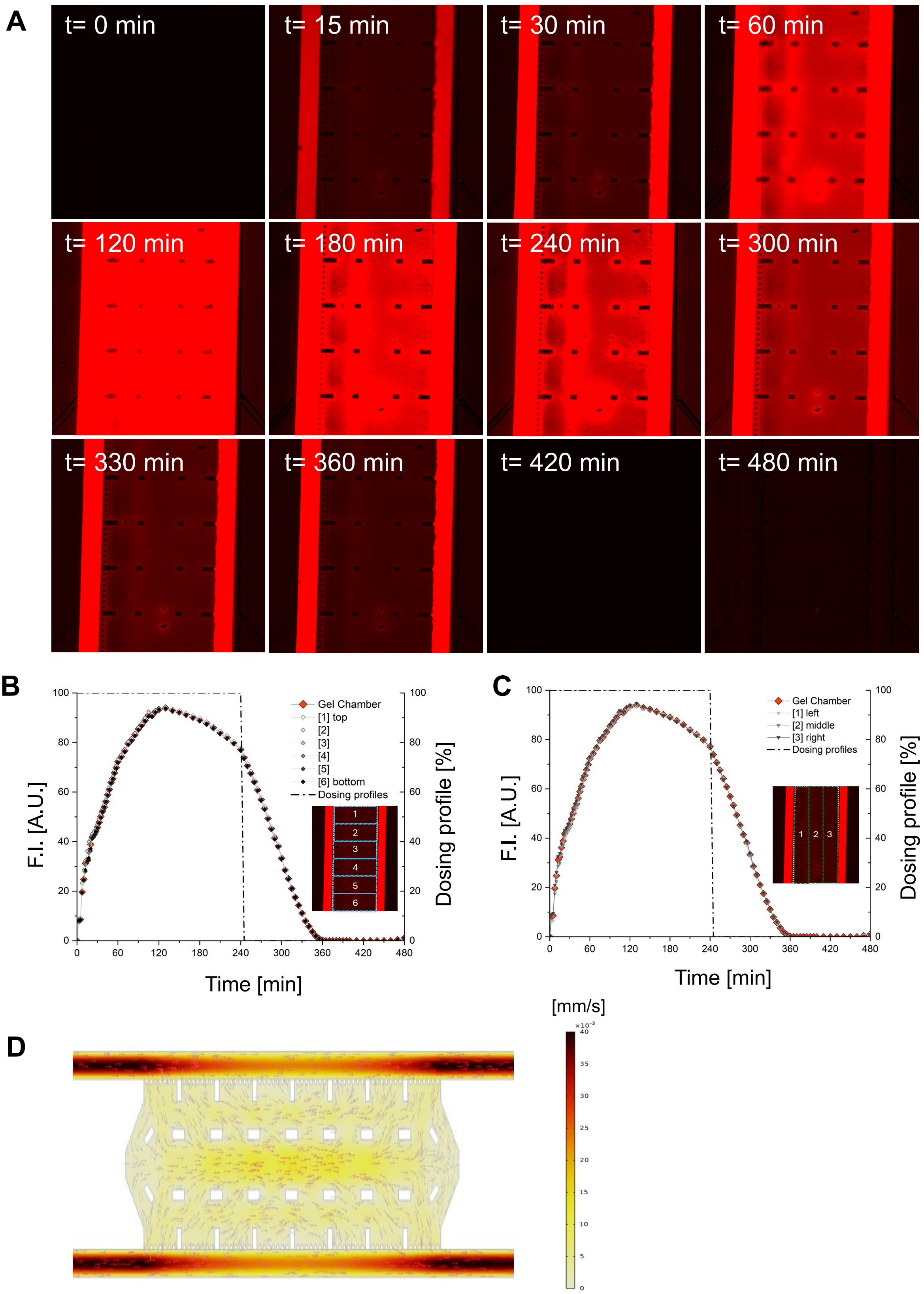

Figure SI 2. *Characterization of the microfluidic platform using sulforhodamine B (SRB) [0.1 mM] diluted in MH medium. (A) Representative micrographs of a 4-hour interval profile (one peak) over 480 min; Confirmation of the homogeneous distribution within the chamber (B) from top to bottom in flow direction and (C) from side channels to middle of the chamber. (D) COMSOL simulation of the flow velocity in the PD chip.*

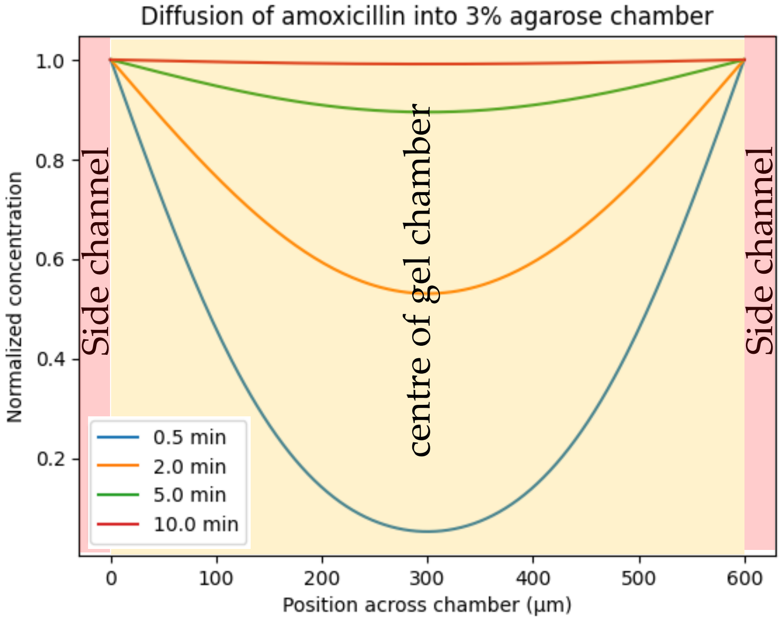

Figure SI 3. *Diffusion of amoxicillin in the central chamber at various time points. After 2–3 minutes amoxicillin has reached the center. 5–10 minutes are required for near-complete homogenization across the gel.*

The diffusion of amoxicillin across the gel was estimated based on the following considerations.

1) The diffusion coefficient of amoxicillin in water can be described by the Stokes–Einstein equation:
$D_0 = \frac{k_B T}{6\pi\eta R_H} = 6 \text{ x } 10^{-10}\ m^2/s$

with $k_B = 1.38 \times 10^{-23}\ J/K$, *temperature* $T = 310\ K$, *viscosity of Water (at 37 °C)* $\eta = 0.0007\ Pa \cdot s$ *and the hydrodynamic radius of amoxicillin with the molecular weight of ≈ 365 Da,* $R_H \approx 0.5 \times 10^{-9}\ m$.

2) The diffusion coefficient in 3 % agarose is reduced by the retardation factor: $f \approx 0.5$ *, accounting for steric hindrance and tortuosity:*
$D_{gel} = D_0 \times f = (6 \times 10^{-10}) \times 0.5 \approx 3 \times 10^{-10}\ m^2/s$

3) The diffusion time to the center of the gel chamber (half width is 300 μm) is defined as follows:
$t = \frac{L^2}{2D_{agarose}} \approx 150\ s$

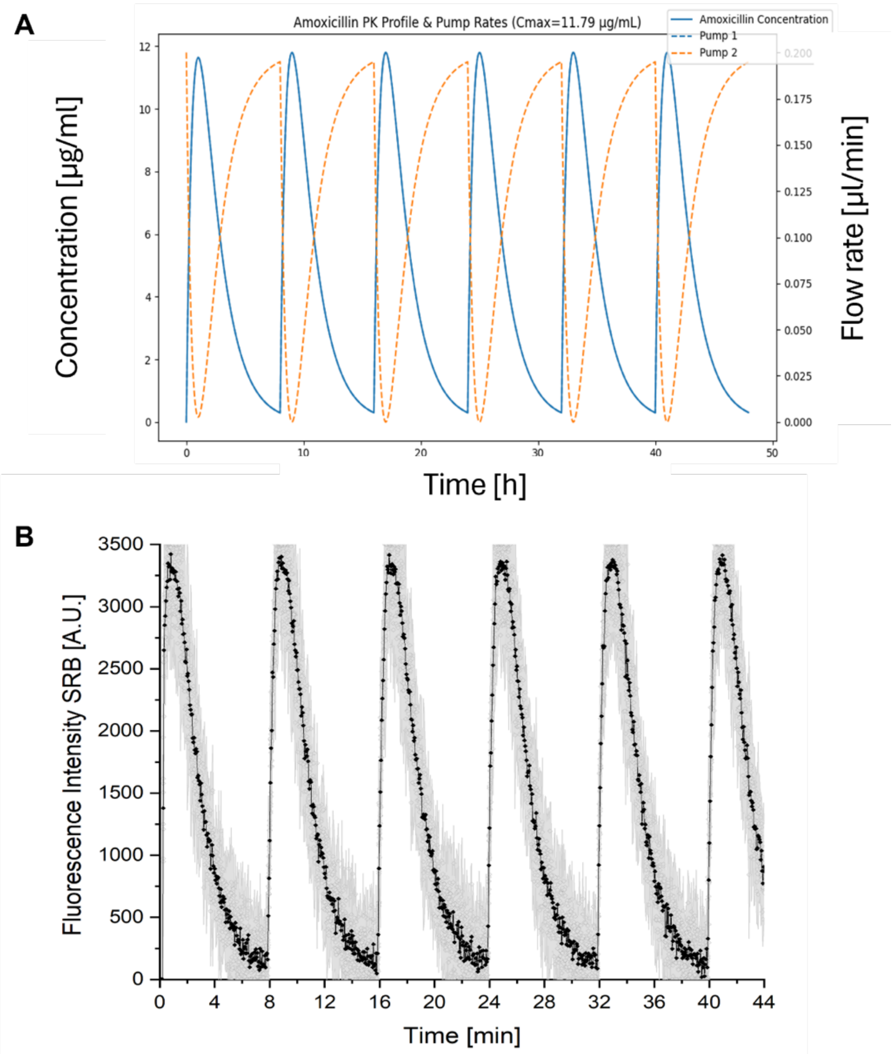

Figure SI 4. *Generation of pharmacokinetic profiles. The programmed pump profile (two pumps: blue: drug; yellow: drug-free medium) in A) result in the profiles in B) depicting the measured outlet concentration determined from sulforhodamine B (SRB) fluorescence intensity over time.*

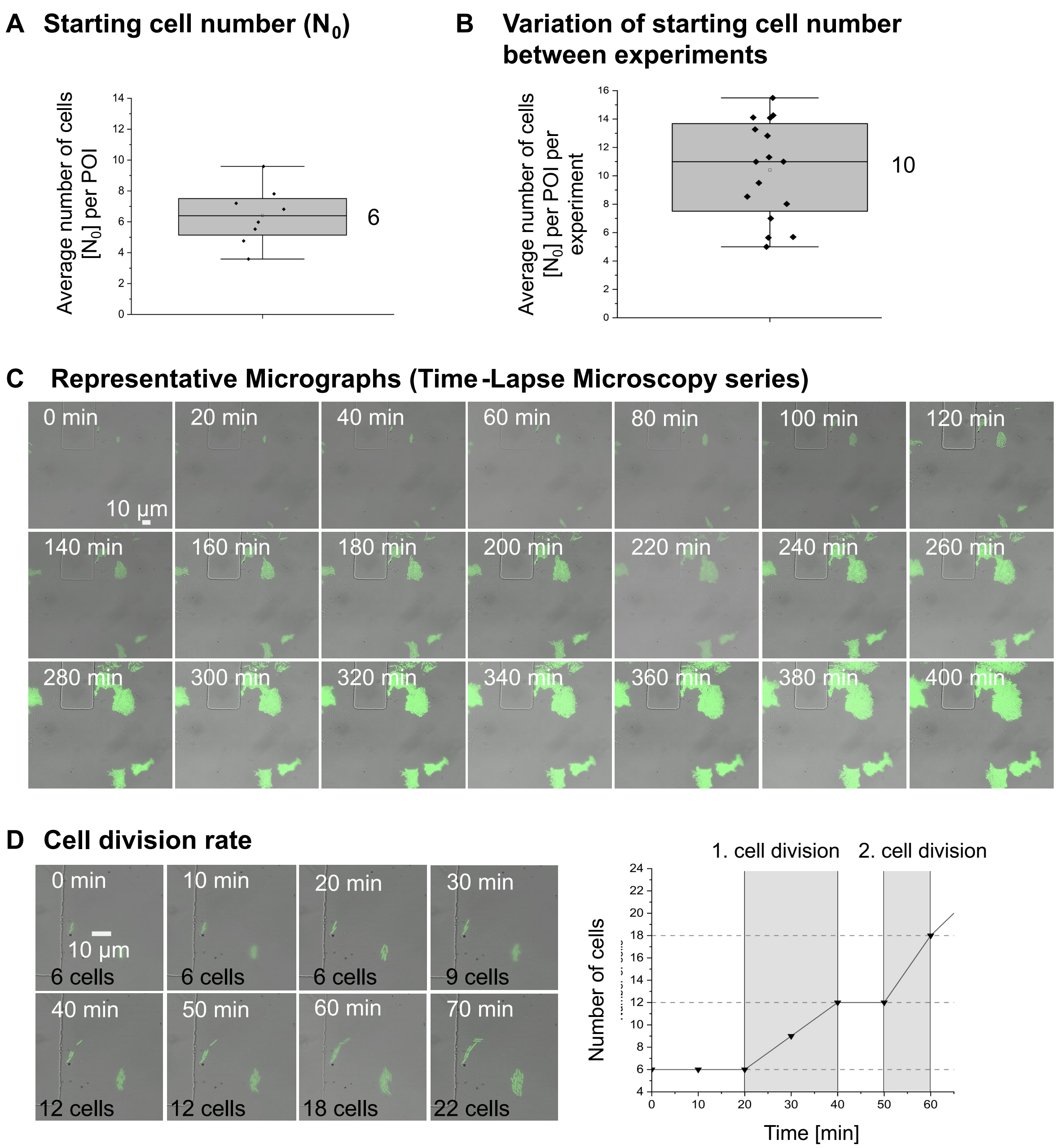

Figure SI 5. *Characterization of bacterial growth of* E. coli *ATCC 25922 in the µHFIM platform. (A) Variation of starting cell number per POI [N=8 chambers, total n=347 POIs] with average starting cell number of 6 cells per POI; (B) Experiment to experiment variation of starting cell number per POI [N=14, total n=6648, every experiment data based on 4 independent chambers]; (C) Representative micrographs over 400 min.; (D) Determination of cell division rate over 60 min analysed from representative micrographs of a time-lapse microscopy series.*

A Image processing workflow

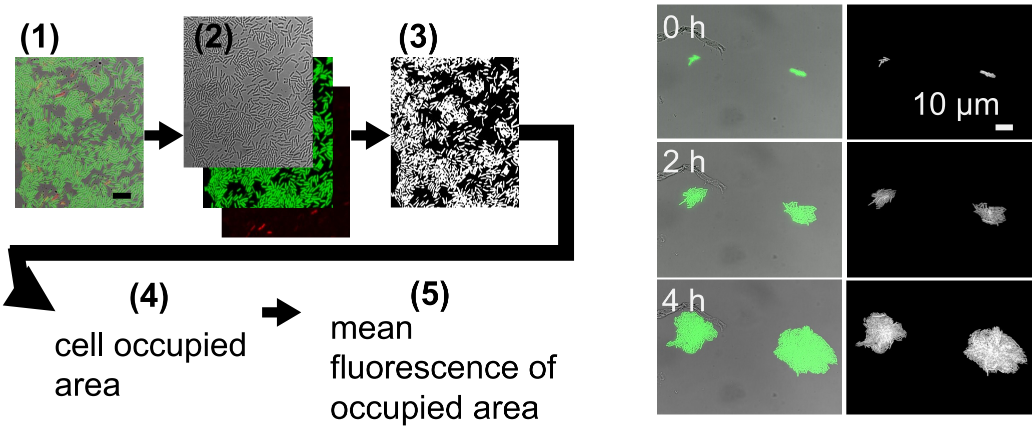

B Mean Fluorescence Intensity (sfGFP)

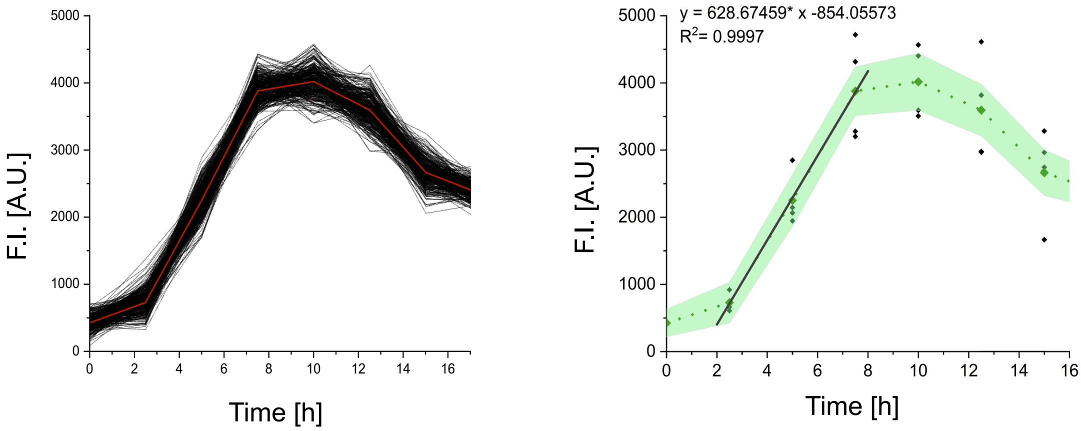

Figure SI 6. *Image processing and bacterial growth of* E. coli *ATCC 25922 in the µHFIM platform. (A) Python Image processing workflow. (1) Multichannel images (.nd2) were transformed into (2) single channel images (.tiff), green and red channels representing live and dead cells, respectively. (3) The image processing pipeline includes thresholding and filtering (otsu). (4) Determination of cell occupied area [µm²] and (5) extraction of mean fluorescence (live and dead cell signal) of occupied area data per POI. Scale bar: 10 µm. (B) Cell viability of* E. coli *ATCC 25922 cells over 16 hours at 37 °C. Growth measured as mean fluorescence intensity of the cell occupied area of expressed sfGFP [N=4 chambers, n=366 POI].*

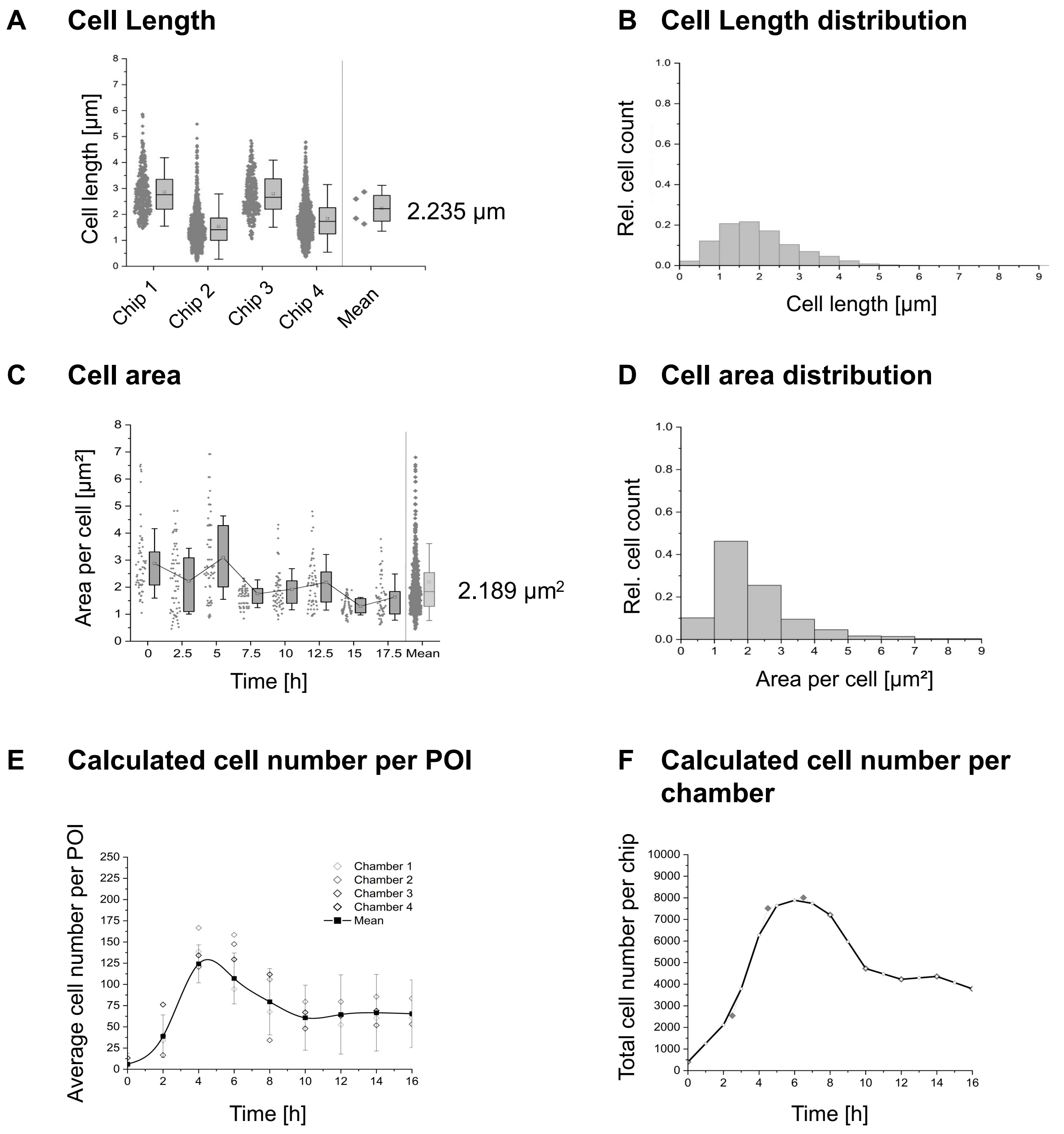

Figure SI 7. *Characterization of bacterial growth based on cell occupied area (here* E. coli *ATCC 25922, no antibiotic supplement). (A) Determination of average bacterial cell length and (B) cell length distribution, [N=4 chambers, n=2706 cells]. (C) Determination of bacterial cell area [n=500 cells] and (D) cell area distribution over all time points. (E) Calculation of total cell number per POI and (F) per chamber over time (cell occupied area divided by average cell area) [N=4, n = 366 POI]. Cell number per POI calculated to be* N_0*=6 cells, per chamber* N_0*=480 cells and* N_{max} *around 7600 cells.*

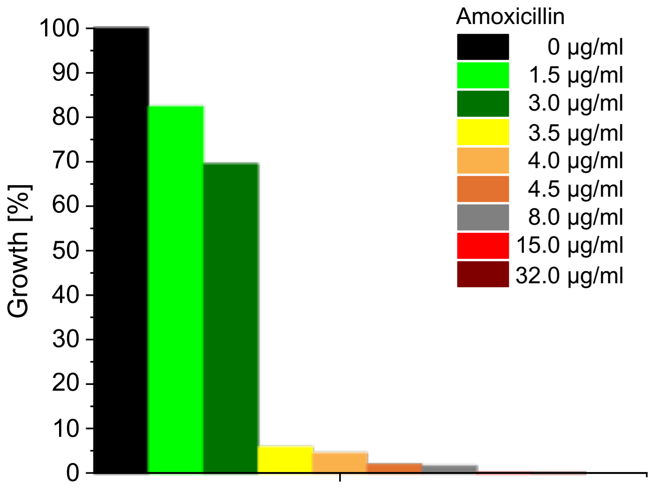

Figure SI 8. *Constant amoxicillin dosing to determine the MIC of* E. coli *ATCC 25922, growth in %, i.e. normalized to the control, after 24 h. MIC between 3 µg/ml and 4 µg/ml.*

Table SI 2: PK/PD indices for interval dosing.

PK parameter								PK/PD indices				
c_{max} *	Cmin	$c_{average}$	fAUC** per peak	fAUC real per peak	Number of peaks	fAUC total	fAUC real total	f c_{max} /MIC	$fc_{average}$ /MIC	fT>MIC	%T> MIC	AUC/MIC
**Constant dosing at MIC [4 µg/ml]**												
4	0	4	-	-	0	64		1	1	0h	0%	1
**2h dosing intervals**												
8	0	4	16	9.47	4	64	37.88	2	1	8h	50%	1
16	0	8	32	18.94	4	128	75.76	4	2	8h	50%	2
24	0	12	48	28.41	4	192	113.64	6	3	8h	50%	3
32	0	16	64	37.88	4	256	151.53	8	4	8h	50%	4
**4h dosing intervals**												
8	0	4	32	31.97	2	64	63.95	2	1	8h	50%	1
16	0	8	64	63.95	2	128	127.90	4	2	8h	50%	2
24	0	12	96	95.92	2	192	191.85	6	3	8h	50%	3
32 l	0	16	128	127.90	2	256	255.80	8	4	8h	50%	4
**8h dosing intervals**												
8	0	4	64	67.34	1	64	67.33	2	1	8h	50%	1
16	0	8	128	134.67	1	128	134.67	4	2	8h	50%	2
24	0	12	192	202.00	1	192	202.00	6	3	8h	50%	3
32	0	16	256	269.34	1	256	269.34	8	4	8h	50%	4

The three most important PK parameters for evaluating antibiotic efficacy are peak concentration (c_{max}), trough concentration (c_{min}), and area under the serum concentration time curve (AUC). These PK/PD indices are the percent of time the free drug concentration remains above the MIC (%fT>MIC), the ratio of the free drug peak concentration to the MIC (f c_{max}:MIC), and the ratio of the area under the free drug concentration time-curve to the MIC (fAUC:MIC) [8]. AUC determination was done using the trapezoidal rule.

*all concentrations in [µg/ml]; **AUC in [(µg*h)/ml]

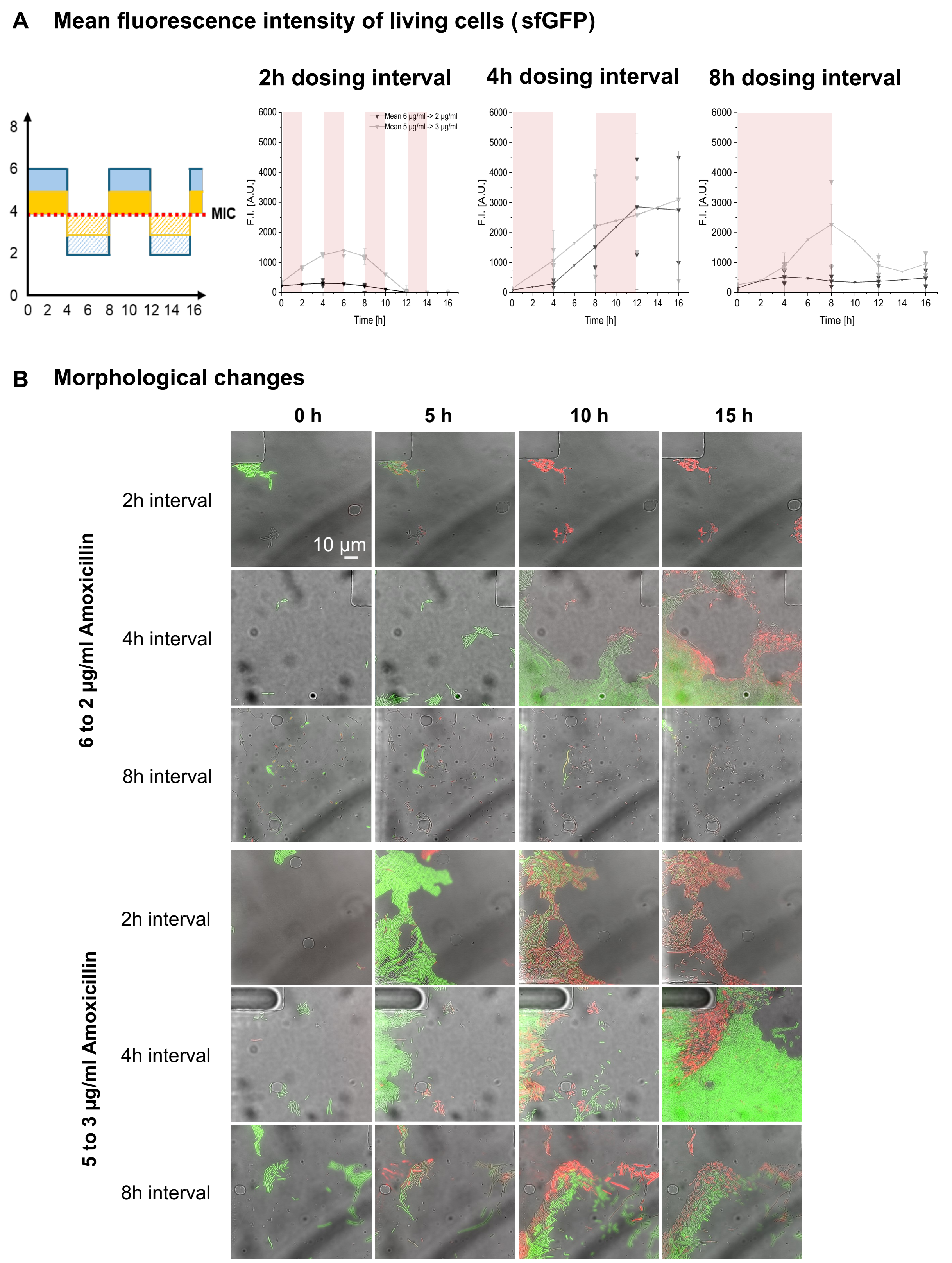

Figure SI 9. *Gradual dosing of amoxicillin around the MIC. (A) Bacterial growth of* E. coli *ATCC 25922 measured over time as mean fluorescence intensity of the cell occupied area per POI under gradual dosing of amoxicillin based on the dosing intervals (2h, 4h, 8h shown from left to right) and different antibiotic concentrations ($c_{average}$= 4 µg/ml) over 16 hours (n = 240 per concentration); (B) Representative micrographs of morphological changes over time.*

Table SI 3: PK/PD indices for gradual dosing around the MIC

Determination of AUC by Trapezoidal rule.

**PK parameter**						**PK/PD indices**						
**c_{max} ***	**Cmin**	**$c_{average}$**	**fAUC** per peak**	**fAUCreal per peak**	**Number of peaks**	**fAUC total**	**fAUCreal total**	**fc_{max}/MIC**	**$fc_{average}$/ MIC**	**$fT_{>MIC}$**	**%T> MIC**	**AUC/ MIC**
Constant dosing at MIC [4 µg/ml]												
4	4	4 l			0	64		1	1	0h	0%	1
2h dosing intervals												
6	2	4	16	9.47	4	64	37.88	2	1	8h	50%	1
5	3	4	16	9.47	4	64	37.88	4	1	8h	50%	1
4h dosing intervals												
6	2	4	32	31.97	2	64	63.95	2	1	8h	50%	1
5	3	4	32	31.97	2	64	63.95	4	1	8h	50%	1
8h dosing intervals												
6	2 l	4	64	67.34	1	64	67.33	2	1	8h	50%	1
5	3	4	64	67.34	1	64	67.33	4	1	8h	50%	1

Determination of AUC by Trapezoidal rule. *Concentration [µg/ml] ; **AUC [(µg*h)/ml]

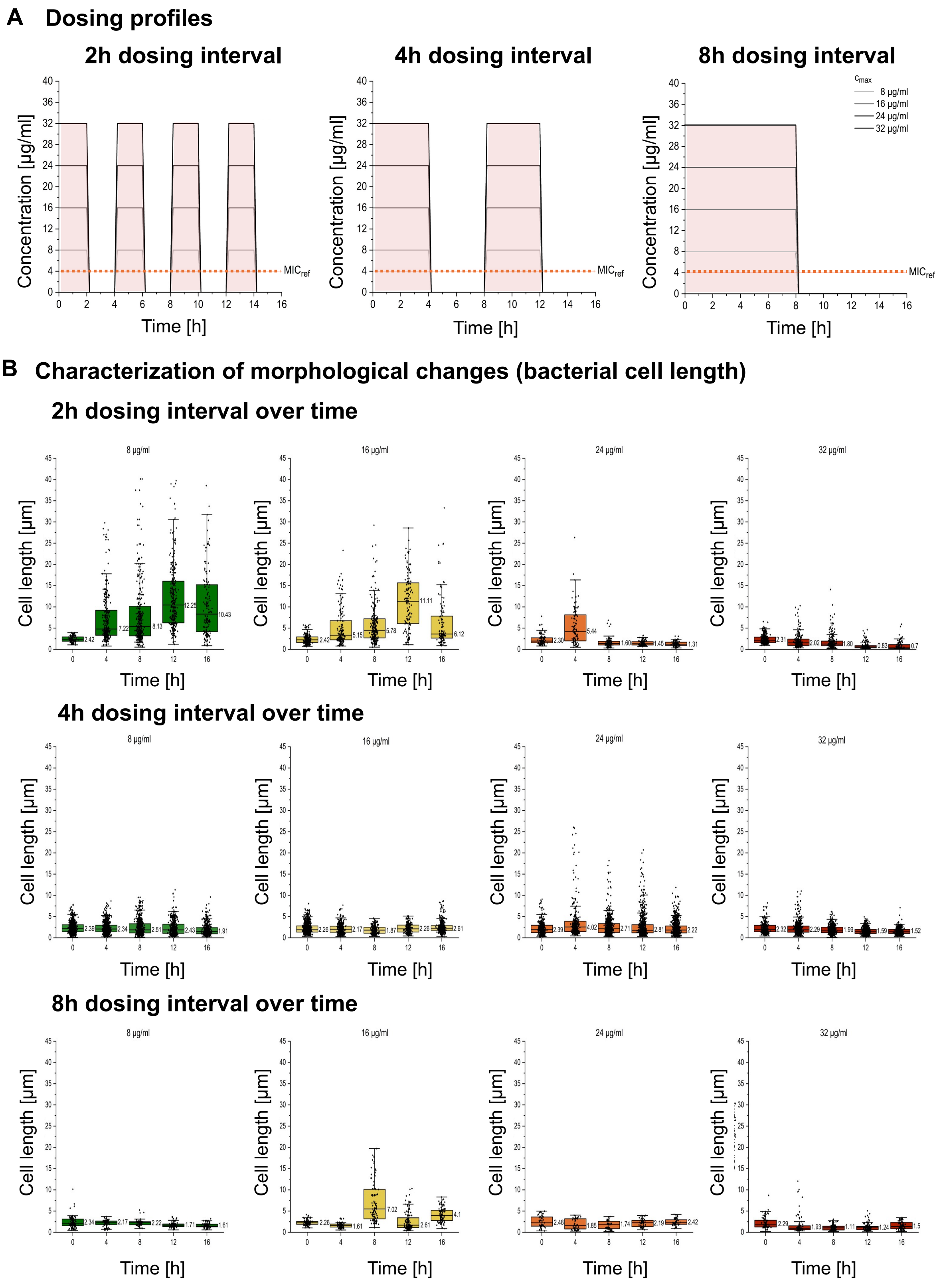

Figure SI 10. *Change of bacterial cell length for different dosing intervals and drug concentrations for* E. coli *ATCC 25922 (sfGFP) cells. (A) Applied dosing profiles. Average amoxicillin concentration equals 50 % of* c_{max}*; (B) Distribution of bacterial cell length of all tested conditions over time [n>100 per time point and condition].*

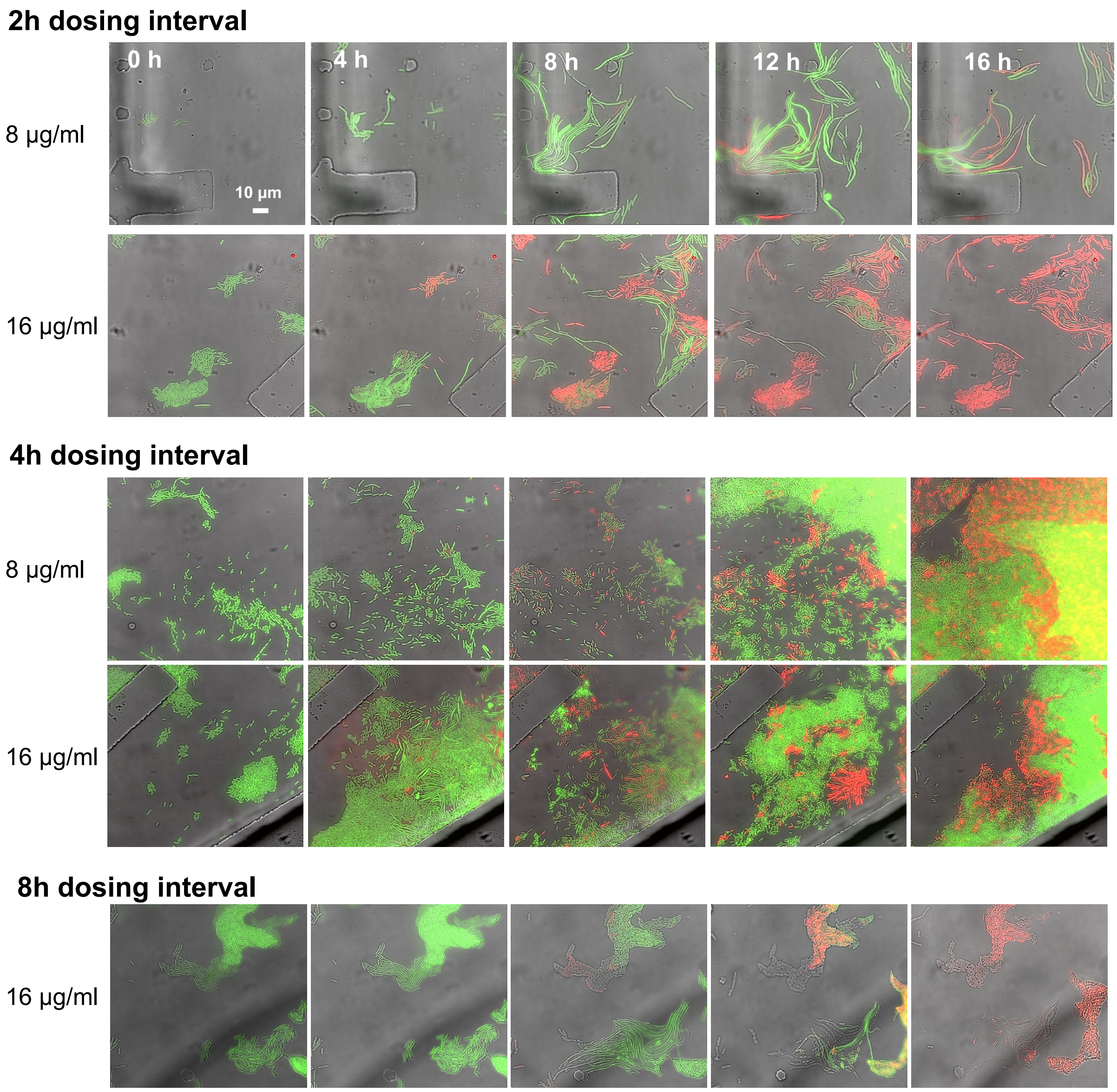

Figure SI 11. *Change of bacterial cell length for different dosing intervals and drug concentrations for* E. coli *ATCC 25922 (sfGFP) cells. Representative micrographs for the 2h-, 4h and 8h intervals, shown in Figure 6A.*

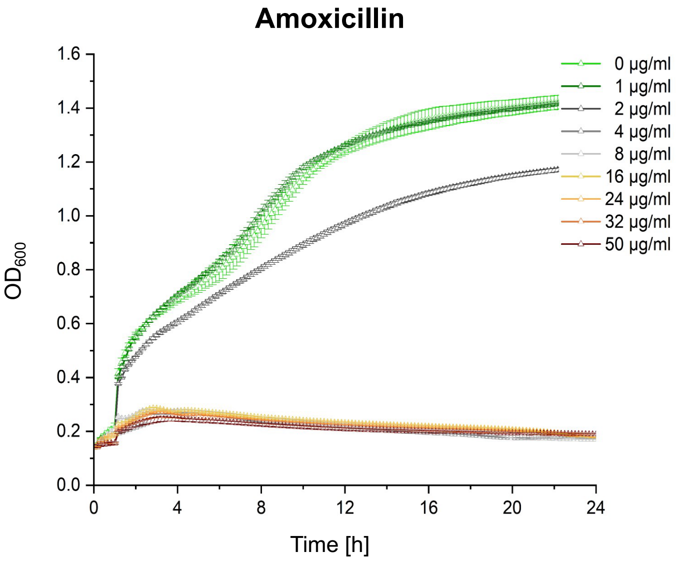

Figure SI 12. *Determination of the MIC for the clinical isolates. Plate reader MIC determination for amoxicillin sensitive* E. coli *clinical isolate 23060725*

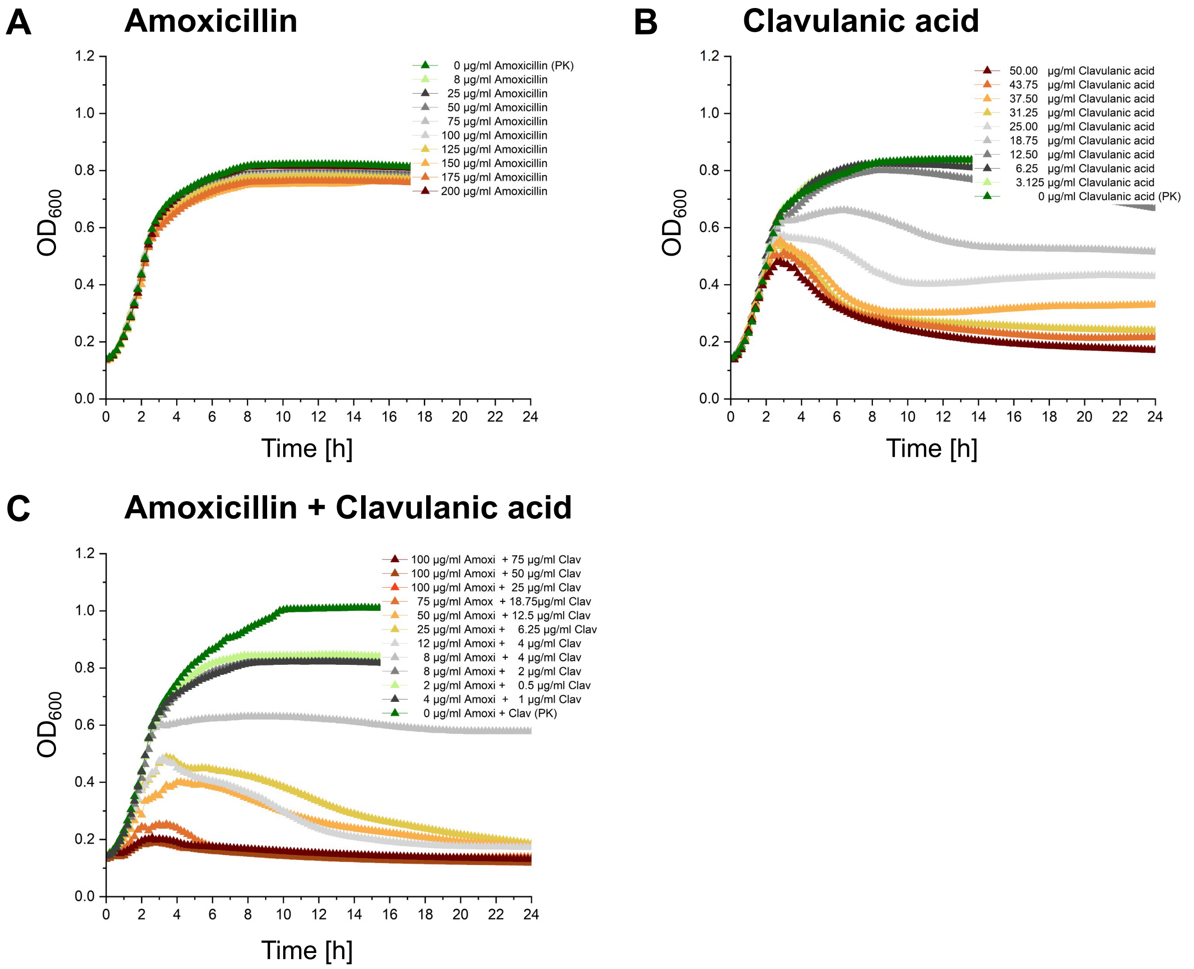

Figure SI 13. *MIC determination of amoxicillin-resistant, sfGFP-producing* E. coli *ATCC 35218 in a plate reader for treatment with (A) amoxicillin, (B) clavulanic acid and (C) amoxicillin combined with Clavulanic acid.*

References

1. Armbrecht, L., Müller, R. S., Nikoloff, J. & Dittrich, P. S. Single-cell protein profiling in microchambers with barcoded beads. *Microsyst Nanoeng* ***5**, 55 (2019).*

2. Jusková, P. et al. Real-Time Respiration Changes as a Viability Indicator for Rapid Antibiotic Susceptibility Testing in a Microfluidic Chamber Array. *ACS Sens* ***6**, 2202–2210 (2021).*

3. Choi, J. et al. Rapid antibiotic susceptibility testing by tracking single cell growth in a microfluidic agarose channel system. *Lab Chip* ***13**, 280–287 (2013).*

4. Clinical and Laboratory Standards Institute (CLSI). CLSI. *Performance Standards for Antimicrobial Susceptibility Testing. 28th ed.* CLSI supplement M100. *(2018).*

5. Stiefel, P., Schmidt-Emrich, S., Maniura-Weber, K. & Ren, Q. Critical aspects of using bacterial cell viability assays with the fluorophores SYTO9 and propidium iodide. *BMC Microbiol* **15**,*36 (2015).*

6. Robertson, J., McGoverin, C., Vanholsbeeck, F. & Swift, S. Optimisation of the protocol for the liVE/DEAD®BacLightTM bacterial viability kit for rapid determination of bacterial load. *Front Microbiol* **10**,*801 (2019).*

7. Ou, F., Mcgoverin, C., Swift, S. & Vanholsbeeck, F. Rapid Evaluation of Bacterial Viability Using the Optrode-a near Real Time Portable Fluorimeter. *Photonics and Fiber Technology 2016 (ACOFT, BGPP, NP), AW3C.6 (2016).*

8. Labreche, M. J., Graber, C. J. & Nguyen, H. M. Recent updates on the role of pharmacokinetics-pharmacodynamics in antimicrobial susceptibility testing as applied to clinical practice. *Clinical Infectious Diseases* **61,** 1446–1452 (2015).
